# Correcting Spatial Variance of RCM for GEO SAR Imaging Based on Time-Frequency Scaling

**DOI:** 10.3390/s16071091

**Published:** 2016-07-14

**Authors:** Ze Yu, Peng Lin, Peng Xiao, Lihong Kang, Chunsheng Li

**Affiliations:** 1 School of Electronics and Information Engineering, Beihang University, Beijing 100191, China; yz613@buaa.edu.cn (Z.Y.); linpeng@buaa.edu.cn (P.L.); lichunsheng@buaa.edu.cn (C.L.); 2 Beijing Institute of Remote Sensing Information, Beijing 100192, China; gregrs@126.com

**Keywords:** geosynchronous synthetic aperture radar, imaging, spatial variance correction, time-frequency scaling

## Abstract

Compared with low-Earth orbit synthetic aperture radar (SAR), a geosynchronous (GEO) SAR can have a shorter revisit period and vaster coverage. However, relative motion between this SAR and targets is more complicated, which makes range cell migration (RCM) spatially variant along both range and azimuth. As a result, efficient and precise imaging becomes difficult. This paper analyzes and models spatial variance for GEO SAR in the time and frequency domains. A novel algorithm for GEO SAR imaging with a resolution of 2 m in both the ground cross-range and range directions is proposed, which is composed of five steps. The first is to eliminate linear azimuth variance through the first azimuth time scaling. The second is to achieve RCM correction and range compression. The third is to correct residual azimuth variance by the second azimuth time-frequency scaling. The fourth and final steps are to accomplish azimuth focusing and correct geometric distortion. The most important innovation of this algorithm is implementation of the time-frequency scaling to correct high-order azimuth variance. As demonstrated by simulation results, this algorithm can accomplish GEO SAR imaging with good and uniform imaging quality over the entire swath.

## 1. Introduction

Geosynchronous synthetic aperture radar (GEO SAR) operates at an altitude ~36,000 km [1]. Compared with a low-Earth orbit (LEO) SAR, greater coverage can be achieved by GEO SAR because of its much higher orbit [2]. Furthermore, GEO SAR can guarantee observation of the same location every 24 h with the same incidence angle, which cannot be realized by LEO SAR [3]. Given its special characteristics, GEO SAR has attracted much attention [4]. It has become part of a global earthquake satellite system to monitor the global seismic state, as proposed by NASA and JPL in 2003 [5]. Another GEO SAR system called Geosynchronous Earth Monitoring by Interferometry and Imaging (GEMINI) was put forward in 2012 to acquire Earth surface data through GEO SAR interferometry [6]. For GEO SAR systems, imaging is always a major problem, the key to which is correction of the spatial variance of range cell migration (RCM).

RCM determines the distribution of echoes in the time and frequency domains. The spatial variance of RCM causes the spectrums corresponding to targets at different locations to be different. Therefore, in order to accomplish precise focusing in the frequency domain, the spatial variance must be corrected to eliminate the difference of RCM between any target in the swath and the reference point, usually the swath center. For LEO SAR with zero-Doppler steering, RCM is spatially variant along the range direction only, which can be processed by algorithms, such as range Doppler (RD) [7], chirp scaling (CS) [8] and wavenumber domain (WD) [9]. For high-squint LEO SAR, RCM varies along both range and azimuth slightly, and can be corrected by azimuth frequency scaling and block processing [10]. Compared with LEO SAR, higher altitude makes the synthetic aperture time of GEO SAR increase by over 100 times, which causes the relative trajectories between SAR and targets to become much more curved. As a result, for GEO SAR, the spatial variance of RCM is not only present in both range and azimuth directions but also much greater and more complicated. Moreover, the better the spatial resolution is, the much more serious the spatial variance of RCM is. How to correct the two-dimensional spatial variance of RCM is the specific imaging issue to realize high-resolution GEO SAR imaging. To address this problem, a precise range model has to be constructed to analyze RCM. Furthermore, an imaging algorithm based on the model can be presented to correct spatial variance and focus echo data.

According to this basic thought, in 2011, Bao et al. proposed a polynomial model to approximate RCM [11]. The following year they proposed a modified chirp scaling algorithm to correct linear spatial variance along the range direction [12]. After applying this algorithm, the quadratic residual RCM persists after correction. For this defect, Hu et al. forwarded a modified non-linear chirp scaling (NCS) algorithm based on correction of the quadratic residual RCM, but this still neglected spatial variance along the azimuth [13]. To correct the first-order azimuth variance, three different techniques were developed. Sun et al. adopted azimuth scaling combined with chirp scaling [14]. Hu et al. proposed a wavenumber-domain imaging algorithm based on modified Stolt interpolation [15]. Ding et al. constructed a fourth-order space-variant slant range model, applied the quadratic factor compensation in the two-dimensional time domain to reduce the variance of the azimuth phase, and adopted NCS to accomplish imaging. Simulation results demonstrated that this algorithm could enlarge the azimuth size of a well-focused image with a moderate resolution [16]. However, these techniques cannot correct higher-order azimuth variance contained in the quadratic and cubic phases, which is also crucial for imaging quality. In 2015, Li et al. proposed a fifth-order slant range model, and corrected quadratic azimuth spatial variance by exploiting azimuth time scaling. This technique focused on azimuth processing, while the range variance was not considered adequately [17].

The goal of this paper is to develop an algorithm that can correct high-order spatial variance of RCM along the range and azimuth directions, and perform GEO SAR imaging with a resolution of 2 m in both the ground cross-range and range directions. The most important innovation of this algorithm is the implementation of time-frequency scaling to correct linear and quadratic azimuth variance.

This paper is structured as follows: Section 2 constructs an echo model based on approximation of the range history, analyzes spatial variance in the time and frequency domain, and proposes explicit expressions for phases in terms of spatial variables. To correct the spatial variance, Section 3 presents the basic methodology. A GEO SAR imaging algorithm is advanced in Section 4, which is composed of five steps, i.e., an initial azimuth scaling, RCM correction (RCMC) & range compression, second azimuth scaling, azimuth focusing, and geometric correction. In Section 5, simulation results are addressed to verify the validity of the proposed algorithm. Section 6 concludes the paper.

## 2. Echo Model and Spatial Variance Analysis

For SAR, after demodulation, the echo corresponding to an isolated point target can be represented by:

(1) $$s_{original}(\eta ,\tau )=\sigma \cdot rect\left[\frac{\eta -\eta_{c}}{T_{s}}\right]\cdot a\left[\tau -\frac{2R_{actual}\left(\eta \right)}{c_{0}}\right]\cdot \exp\left[-j\frac{4\pi }{\lambda }R_{actual}\left(\eta \right)\right]$$

where η and τ denote the slow time along the azimuth and the fast time along the range, respectively. The constant σ is the backscattering coefficient of the target, $c_{0}$ is light speed, and λ is wavelength. a(τ) represents the transmitted signal. Here, the chirp signal is the transmitted signal, which implies $a\left(\tau \right)=rect\left[\tau /T_{p}\right]\exp\left[j\pi K_{r}\tau^{2}\right]$. $K_{r}$ is the linear frequency modulation rate, $T_{p}$ is pulse duration, and rect[⋅] denotes the rectangular envelope. $\eta_{c}$ is the beam crossing time and $T_{s}$ is the synthetic aperture time.

In Equation (1), $R_{actual}\left(\eta \right)$ is the equivalent slant range between the spaceborne SAR and the target, which is the average of the one-way slant range when transmitting a pulse and that when receiving the echo. Usually $R_{actual}\left(\eta \right)$ can be approximated by a polynomial:

(2) $$R_{actual}\left(\eta \right)\approx R\left(\eta \right)=\sum_{n=0}^{N_{r}}\frac{r_{n}}{n!}{\left(\eta -\eta_{c}\right)}^{n}$$

where $r_{n}$ is the *n*th-order coefficient, and $N_{r}$ denotes the order. All SAR imaging algorithms are derived based on an appropriate slant range model, like Equation (2). The higher the order, the better the fit, which leads to better imaging performance. However, a high order increases the complexity of imaging algorithms. Therefore, determining an $N_{r}$ that balances imaging quality and complexity of the algorithm is a major challenge.

Usually the aperture time of LEO SAR is so short that Equation (2) with $N_{r}=2$ is adequate to approximate the slant range, which indicates that the phase error induced by the approximation is too small to affect imaging quality [18]. For GEO SAR, orbital altitude increases the aperture time over one hundred times. For example, if the ground resolution is 2 m, the aperture times for LEO and GEO SARs are about 6 s and 750 s, respectively. Thus, to describe the much more complicated relative motion with the longer aperture time, the order of Equation (2) must be redetermined by evaluating the impact of different $N_{r}$ on imaging quality, which can be represented by the resolution, peak side-lobe level ratio (PSLR), and integral side-lobe level ratio (ISLR) [18].

The following matching filtering is adopted:

(3) $$s_{opt}\left(\eta ,\tau \right)=\exp\left[-j\frac{4\pi }{\lambda }R_{actual}\left(\eta \right)\right]⊗\exp\left[j\frac{4\pi }{\lambda }R\left(-\eta \right)\right]$$

where ⊗ represents convolution and $N_{r}$ may equal 4, 5 or ∞. When $N_{r}$ approaches ∞, Equation (3) indicates ideal filtering, which can achieve optimal imaging quality per the theory of matching filtering [18].

**Figure 1:**
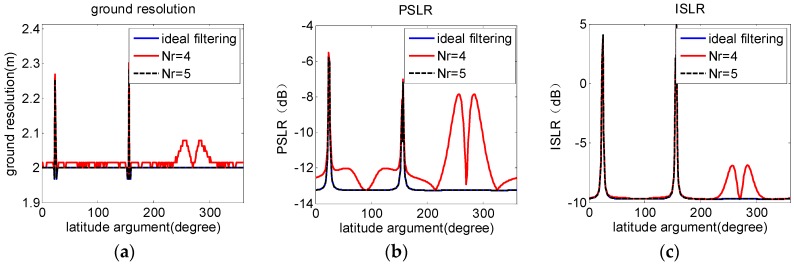
Comparison of ideal filtering and matching filtering with $N_{r}=$ 4 and 5. (**a**–**c**) show results of azimuthal resolution, PSLR, and ISLR during the entire orbital period, respectively. Results achieved with $N_{r}=5$ are as nearly identical to those attained by ideal filtering. With $N_{r}=4$ , results are much worse.

Evaluation results of applying Equation (3) and parameters in Table 1 are shown in Figure 1. Azimuthal resolution, PSLR, and ISLR achieved with $N_{r}=5$ are as nearly identical to results of the ideal filtering during the entire orbital period. However, when $N_{r}=4$, results are much worse. Therefore, the fifth-order approximation to $R_{actual}(\eta )$ is adequate to acquire optimum imaging quality.

By combining Equations (1) and (2), the echo model can be expressed as:

(4) $$s_{original}(\eta ,\tau )\approx \sigma \cdot rect\left[\frac{\eta -\eta_{c}}{T_{s}}\right]\cdot \exp\left\{j\pi K_{r}{\left[\tau -\frac{2R(\eta )}{c_{0}}\right]}^{2}\right\}\cdot \exp\left\{-j\frac{4\pi }{\lambda }R(\eta )\right\}$$

### 2.1. Spatial Variance in the Time Domain

Given the invalidity of the “stop-and-go” approximation, $r_{n}$ (n≥0) in Equation (2) can be expressed as follows (see Appendix A):

(5) $$r_{n}=r_{n,\sin}-\frac{k_{n+1}}{c_{0}}-\frac{1}{c_{0}^{2}}\cdot \sum_{m=0}^{n}C_{n}^{m}k_{m+2}r_{n-m,\sin},n\geq 0$$

where:

$$k_{n}=\langle {{\vec{R}}_{g\_sat}^{\left(n\right)}|}_{\eta =\eta_{c}},{\vec{R}}_{g\_tar}^{\left(n\right)}\rangle$$

and 〈⋅〉 denotes the dot product. $C_{n}^{m}=n!/\left[(n-m)!m!\right]$ and $r_{n,\sin}$ denotes the *n*th-order one-way slant range coefficient. ${{\vec{R}}_{g\_sat}^{\left(n\right)}|}_{\eta =\eta_{c}}$ represents *n*th-order derivatives of the position vectors of the SAR satellite at the beam crossing time $\eta_{c}$ and ${\vec{R}}_{g\_tar}^{\left(n\right)}$ represents the position vector of the target. Usually the attitude steering is applied for GEO SAR [19], and makes the Doppler centroid zero, which leads to $r_{1}=0$.

**Figure 2:**
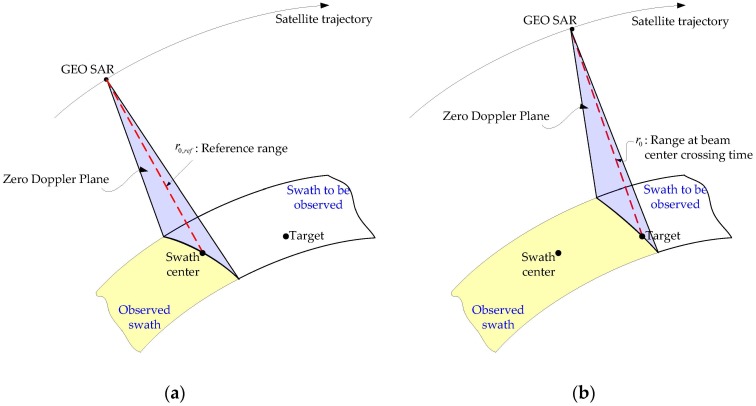
Illustration of GEO SAR geometry. (**a**) Observation geometry when the beam crosses the swath center; (**b**) Observation geometry when the beam crosses another target.

Figure 2 illustrates the GEO SAR observation geometry. The beam crossing time corresponding to a target is the moment when the zero Doppler plane crosses the target. At the beam crossing time corresponding to the swath center, the distance from GEO SAR to the swath center is defined as the reference range, i.e., $r_{0,ref}$, as shown in Figure 2a. When the zero Doppler plane crosses any target in the swath at $\eta_{c}$, the distance from SAR to the target is denoted by $r_{0}$, as shown in Figure 2b. As a result, the location of an isolated target can be uniquely represented by the beam crossing time $\eta_{c}$ and the corresponding distance $r_{0}$.

Equation (5) demonstrates that $r_{n}$ depends on the target position, indicating that $r_{n}$ is spatially variant. Therefore, using polynomial fitting, $r_{n}$ can be expressed as a function of ΔR and $\eta_{c}$, which are adopted as spatial variables along the range and azimuth directions, respectively. That is:

(6) $$\left\{ \begin{array}{l} r_{0}=r_{0,ref}+\Delta R\\ r_{1}=0\\ r_{2}=r_{2,ref}+k_{2,1,r}\cdot \Delta R+k_{2,2,r}\cdot {\left(\Delta R\right)}^{2}+k_{2,1,a}\cdot \eta_{c}+k_{2,2,a}\cdot {\left(\eta_{c}\right)}^{2}\\ r_{3}=r_{3,ref}+k_{3,1,r}\cdot \Delta R+k_{3,1,a}\cdot \eta_{c}+k_{3,2,a}\cdot {\left(\eta_{c}\right)}^{2}\\ r_{4}=r_{4,ref}+k_{4,1,r}\cdot \Delta R+k_{4,1,a}\cdot \eta_{c}\\ r_{5}=r_{5,ref} \end{array}\right.$$

where $\Delta R=r_{0}-r_{0,ref}$. $r_{n,ref}$, $k_{n,m,a}$ and $k_{n,m,r}$ can be calculated by polynomial fitting based on ephemeris data and geographic information of the swath. All coefficients are spatially invariant except $k_{n,m,a}$, which varies with ΔR. Equation (6) demonstrates that there is nonlinear spatial variance in the slant range coefficients, which leads to the same condition in the phase spectrum.

### 2.2. Spatial Variance in the Frequency Domain

Equation (6) demonstrates that coefficient $r_{n}$ is two-dimensionally spatially variant, and so does RCM in the time domain. By implementing the Fourier transform (FT) on $s_{original}\left(\eta ,\tau \right)$ in range, the signal in the range-frequency domain can be expressed as follows:

(7) $$S_{range\_fft}\left(\eta ,f_{\tau }\right)=\sigma \cdot rect\left[\frac{\eta -\eta_{c}}{T_{s}}\right]\cdot rect\left[\frac{f_{\tau }}{B_{r}}\right]\exp\left\{-j\frac{4\pi \left(f_{0}+f_{\tau }\right)}{c_{0}}R\left(\eta \right)\right\}\exp\left\{-j\pi \frac{f_{\tau }^{2}}{K_{r}}\right\}$$

where $f_{\tau }$ is range frequency, $B_{r}$ is bandwidth of the transmitted signal, and $f_{0}$ is the carrier frequency. Then by performing FT along the azimuth, the two-dimensional spectrum is as follows (see Appendix B):

(8) $$S_{2df}\left(f_{\eta },f_{\tau }\right)=\sigma \cdot W_{a}\left[f_{\eta }\right]\cdot rect\left[\frac{f_{\tau }}{B_{r}}\right]\cdot \exp\left\{-j\frac{4\pi \left(f_{0}+f_{\tau }\right)}{c_{0}}\left[r_{0}-\sum_{n=2}^{10}\frac{A_{n-1}}{n}{\left(-\frac{c_{0}f_{\eta }}{2\left(f_{0}+f_{\tau }\right)}-r_{1}\right)}^{n}\right]\right\}\cdot \exp\left\{-j\frac{\pi f_{\tau }^{2}}{K_{r}}-j2\pi f_{\eta }\eta_{c}\right\}$$

where $A_{n}$ is defined as follows:

(9) $$\left\{ \begin{array}{l} A_{1}=\frac{1}{r_{2}}\\ A_{2}=-\frac{r_{3}}{2r_{2}^{3}}\\ A_{3}=\frac{1}{6r_{2}^{5}}\left[3r_{3}^{2}-r_{2}r_{4}\right]\\ A_{4}=\frac{1}{24r_{2}^{7}}\left[10r_{2}r_{3}r_{4}-15r_{3}^{3}-r_{2}^{2}r_{5}\right]\\ A_{5}=\frac{1}{120r_{2}^{9}}\left[-r_{2}^{3}r_{6}+15r_{5}r_{2}^{2}r_{3}+10r_{2}^{2}r_{4}^{2}-105r_{2}r_{3}^{2}r_{4}+105r_{3}^{4}\right]\\ A_{6}=\cdots \end{array}\right.$$

$W_{a}\left[f_{\eta }\right]$ is the azimuth spectrum amplitude. Since it is only concerned with azimuth focusing, its representation will be given in Section 4. By applying series expansion [20], Equation (8) can be organized in the form of a series of $f_{\tau }$, which is:

(10) $$S_{2df}\left(f_{\eta },f_{\tau }\right)=\sigma \cdot W_{a}\left[f_{\eta }\right]\cdot rect\left[\frac{f_{\tau }}{B_{r}}\right]\cdot \exp\left\{j2\pi \sum_{k=1}^{9}\phi_{k}f_{\tau }^{k}\right\}\cdot \exp\left\{j2\pi \varphi_{0}\right\}\exp\left\{-j2\pi f_{\eta }\eta_{c}\right\}$$

where:

(11) $$\left\{ \begin{array}{l} \varphi_{0}=-\frac{2}{\lambda }\left(r_{0}-P_{0}\right)+\sum_{m=1}^{10}\frac{2P_{m}}{\lambda }{\left(\frac{\lambda }{2}f_{\eta }\right)}^{m}\\ \phi_{1}=-\frac{2}{c_{0}}\left(r_{0}-P_{0}\right)-\sum_{m=2}^{10}\frac{2P_{m}}{c_{0}}{\left(\frac{\lambda }{2}f_{\eta }\right)}^{m}\left(m-1\right)\\ \phi_{2}=\sum_{m=2}^{10}\frac{2C_{m}^{2}P_{m}}{c_{0}f_{0}}{\left(\frac{\lambda }{2}f_{\eta }\right)}^{m}-\frac{1}{2K_{r}}\\ \phi_{k}={\left(-1\right)}^{k}\sum_{m=2}^{10}\frac{2C_{m+k-2}^{k}P_{m}}{c_{0}f_{0}^{k-1}}{\left(\frac{\lambda }{2}f_{\eta }\right)}^{m},k\geq 3 \end{array}\right.$$

(12) $$\left\{ \begin{array}{l} P_{0}=\sum_{n=1}^{9}\frac{A_{n}{\left(-r_{1}\right)}^{n+1}}{n+1}\\ P_{1}=\sum_{n=1}^{9}A_{n}{\left(-1\right)}^{n+1}r_{1}^{n}\\ P_{m}=\sum_{n=m-1}^{9}\frac{A_{n}{\left(-1\right)}^{n+1}C_{n+1}^{m}r_{1}^{n+1-m}}{n+1},m\geq 2 \end{array}\right.$$

$\varphi_{0}$, $\phi_{1}$, $\phi_{2}$ and $\phi_{k}$ denote the azimuth modulation phase, RCM, range linear frequency-modulated phase and high-order range frequency-modulated phase, respectively. Equations (9), (11) and (12) demonstrate that $\varphi_{0}$ and $\phi_{k}$ depend on $r_{n}$. Therefore, $\varphi_{0}$ and $\phi_{k}$ are spatially variant, which can also be explicitly expressed as a function of ΔR and $\eta_{c}$:

(13) $$\left\{ \begin{array}{l} \varphi_{0}\approx \varphi_{0,ref}+M_{1,r}\Delta R+M_{2,r}{\left(\Delta R\right)}^{2}+M_{1,a}\eta_{c}+M_{2,a}\eta_{c}^{2}\\ \phi_{1}\approx \phi_{1,ref}+L_{1,r}\Delta R+L_{2,r}{\left(\Delta R\right)}^{2}+L_{1,a}\eta_{c}+L_{2,a}\eta_{c}^{2}\\ \phi_{2}\approx \phi_{2,ref}+J_{1,r}\Delta R+J_{1,a}\eta_{c}+J_{2,a}\eta_{c}^{2}\\ \phi_{3}\approx \phi_{3,ref}+K_{1,r}\Delta R+K_{1,a}\eta_{c}\\ \phi_{k}\approx \phi_{k,ref},4\leq k\leq 9 \end{array}\right.$$

All coefficients in Equation (13) depend on $f_{\eta }$, and some of them also vary with ΔR. Details are given in Appendix B. The phase difference between Equations (11) and (13) is <0.012π, indicating that these approximations in Equation (13) will not affect imaging quality.

## 3. Basic Methodology of Correcting Spatial Variance

According to Equation (2), RCM is determined by slant range coefficients (i.e., $r_{n}$). In order to correct the spatial variance of RCM, this paper adopts time-frequency scaling to modify $r_{n}$.

To introduce this idea, a one-dimensional signal s(t) is assumed to be:

(14) $$s\left(t\right)=rect\left[\frac{t-t_{c}}{T}\right]\exp\left\{-j\frac{4\pi }{\lambda }\left[d_{0}+\sum_{n=2}^{N}\frac{d_{n}}{n!}{\left(t-t_{c}\right)}^{n}\right]\right\}$$

where T is the signal duration time. $t_{c}$ is the center time and $d_{n}$ is the *n*th-order time-domain phase coefficient. According to Equation (6), $d_{n}$ can be assumed quadratically variant with $t_{c}$. The aim of the time-frequency scaling method is to remove the spatial variance in $d_{n}$, which indicates that $d_{n}$ doesn’t depend on $t_{c}$ after scaling.

By applying series reversion method [21], the corresponding frequency-domain spectrum of Equation (14) can be attained as:

(15) $$S\left(f\right)=\exp\left[j2\pi \sum_{n=2}^{M}D_{n-1}f^{n}\right]\exp\left[-j2\pi ft_{c}\right]$$

$D_{n}$ is the frequency-domain spectrum phase coefficient and can be obtained by applying the method in Appendix B. In order to avoid time-domain aliasing, $D_{1}$ will not be modified by scaling.

Modification of frequency-domain spectrum phase coefficients leads to modification of time-domain phase coefficients. As a result, the following frequency scaling function can be used:

(16) $$H_{frq,scl}\left(f\right)=\exp\left[j2\pi \sum_{n=3}^{M}Z_{n}\cdot f^{n}\right]$$

After multiplication by Equation (16), Equation (15) becomes:

(17) $$S^{\prime }\left(f\right)=\exp\left\{j2\pi \left[D_{1}f^{2}+\sum_{n=3}^{M}\left(D_{n-1}+Z_{n}\right)f^{n}\right]\right\}\exp\left[-j2\pi ft_{c}\right]$$

By applying series reversion method to Equation (17), the following expression can be obtained:

(18) $$s^{\prime }\left(t\right)=\exp\left[-j\frac{4\pi }{\lambda }\Psi \left(t\right)\right]$$

where:

(19) $$\Psi \left(t\right)=d_{0}^{\prime }+\sum_{n=1}^{N}\frac{d_{n}^{\prime }}{n!}{\left(t-t_{c}\right)}^{n}$$

$d_{n}^{\prime }$ is the reconstructed *n*th-order time-domain phase coefficient. Like $d_{n}$, $d_{n}^{\prime }$ is also spatially variant with $t_{c}$. According to Equation (6), spatial variance is up to the second order along both the range and the azimuth. As a result, $d_{n}^{\prime }$ can be assumed a second-order function of $t_{c}$, i.e.,

(20) $$d_{n}^{\prime }=d_{n,ref}^{\prime }+{\frac{\partial d_{n}^{\prime }}{\partial t_{c}}|}_{t_{c}=0}\cdot t_{c}+\frac{1}{2}\cdot {\frac{\partial^{2}d_{n}^{\prime }}{\partial^{2}t_{c}}|}_{t_{c}=0}\cdot t_{c}^{2}$$

where $d_{n,ref}^{\prime }$ corresponds to the swath center. Then, the spatial difference between $d_{n}^{\prime }$ and $d_{n,ref}^{\prime }$ is:

(21) $$\Delta d_{n}^{\prime }=d_{n}^{\prime }-d_{n,ref}^{\prime }\approx {\frac{\partial d_{n}^{\prime }}{\partial t_{c}}|}_{t_{c}=0}\cdot t_{c}+\frac{1}{2}\cdot {\frac{\partial^{2}d_{n}^{\prime }}{\partial^{2}t_{c}}|}_{t_{c}=0}\cdot t_{c}^{2}$$

In order to eliminate the first-order spatial variance of $d_{n}^{\prime }$, the time scaling function to be multiplied with Equation (18) is designed as:

(22) $$H_{n}\left(t\right)=\exp\left[j\frac{4\pi }{\lambda }{\cdot \frac{\partial d_{n}^{\prime }}{\partial t_{c}}|}_{t_{c}=0}\cdot \frac{1}{\left(n+1\right)!}t^{n+1}\right]$$

By multiplying Equations (18) and (22), the linear component of $\Delta d_{n}^{\prime }$ will be removed. Therefore, for correcting the linear spatial variance of all $d_{n}^{\prime }$ (n≥2), the complete time scaling function is:

(23) $$H_{total}\left(t\right)=\exp\left\{j\frac{4\pi }{\lambda }\sum_{n=2}^{N}\left[{\frac{\partial d_{n}^{\prime }}{\partial t_{c}}|}_{t_{c}=0}\cdot \frac{1}{\left(n+1\right)!}t^{n+1}\right]\right\}$$

After multiplying Equation (18) with Equation (23), the signal becomes:

(24) $$s^{\prime \prime }\left(t\right)=\exp\left\{-j\frac{4\pi }{\lambda }\Psi^{\prime }\left(t\right)\right\}$$

where:

(25) $$\Psi^{\prime }\left(t\right)=d_{0}^{\prime \prime }+\sum_{n=2}^{N}\frac{d_{n}^{\prime \prime }}{n!}{\left(t-t_{c}\right)}^{n}$$

The new time-domain phase coefficient is:

(26) $$\left\{ \begin{array}{l} d_{n}^{\prime \prime }=d_{n,ref}^{\prime }+\frac{1}{2}\left[{\frac{\partial^{2}d_{n}^{\prime }}{\partial^{2}t_{c}}|}_{t_{c}=0}-{\frac{\partial d_{n+1}^{\prime }}{\partial t_{c}}|}_{t_{c}=0}\right]\cdot t_{c}^{2},0\leq n\leq N-1\\ d_{N}^{\prime \prime }=d_{N,ref}^{\prime }+\frac{1}{2}\cdot {\frac{\partial^{2}d_{N}^{\prime }}{\partial^{2}t_{c}}|}_{t_{c}=0}\cdot t_{c}^{2} \end{array}\right.$$

Similar to $d_{n}^{\prime }$, $d_{n}^{\prime \prime }$ also varies with $t_{c}$. By applying the series reversion method to Equation (24), the spectrum is:

(27) $$S^{\prime \prime }\left(f_{\eta }\right)=\exp\left\{-j\frac{4\pi }{\lambda }\sum_{m=2}^{M}B_{m}f^{m}\right\}\exp\left[-j2\pi ft_{c}\right]$$

where $B_{m}$ can be acquired by the method in Appendix B.

Because the time scaling has removed the linear spatial variance, $B_{m}$ satisfies:

(28) $${\frac{\partial B_{m}}{\partial t_{c}}|}_{t_{c}=0}=0,\;m\geq 0$$

In order to remove the quadratic spatial variance in $B_{m}$ (m=2,3), the following equation should be satisfied by assigning the appropriate value of $Z_{n}$:

(29) $${\frac{\partial^{2}B_{m}}{\partial {t_{c}}^{2}}|}_{t_{c}=0}=0,\;m=2,3$$

According to Equation (13), $\phi_{k}$ (k>3) is spatially invariant in GEO SAR imaging. And after time-frequency scaling, the linear and quadratic spatial variance has been removed from $B_{m}$ (m=2,3), as shown in Equations (28) and (29). Therefore, focusing for the whole swath can be accomplished in the frequency domain. Although the time-frequency scaling is only applied to correct the azimuth variance in this section, it can also be applied for the range variance correction, as shown in Section 4.

## 4. Spatial Variance Correction and GEO SAR Imaging

Based on the basic idea in Section 3, a GEO SAR imaging algorithm composed of five steps is proposed. The first is to eliminate linear azimuth variance of $\phi_{k}$ through the first azimuth time scaling. The second is to achieve RCM correction and range compression. The third is to correct residual azimuth variance by the second azimuth time-frequency scaling. The fourth and final steps are to accomplish azimuth focusing and correct geometric distortion.

### 4.1. First Azimuth Time Scaling

In Equation (10), $\phi_{k}$ determines the coupling between range and azimuth. The first step applies the time scaling to remove linear azimuth variance of $\phi_{k}$ and guarantees the quality of RCM correction and range compression.

In the range-frequency and azimuth-time domain, the echo can be expressed as Equation (7). The first azimuth scaling function is designed as:

(30) $$H_{AS1}\left(\eta ,f_{\tau }\right)=\exp\left\{-j\frac{4\pi \left(f_{0}+f_{\tau }\right)}{c_{0}}r_{pt}\left(\eta \right)\right\}$$

where:

(31) $$r_{pt}\left(\eta \right)=-\sum_{m=2}^{4}\frac{{k_{m,1,a}|}_{\Delta R=0}}{\left(m+1\right)!}\eta^{m+1}$$

After multiplying Equations (7) and (30), the signal becomes:

(32) $$S_{AS1}\left(\eta ,f_{\tau }\right)=\sigma \cdot rect\left[\frac{\eta -\eta_{c}}{T_{s}}\right]\cdot rect\left[\frac{f_{\tau }}{B_{r}}\right]\exp\left\{-j\frac{4\pi \left(f_{0}+f_{\tau }\right)}{c_{0}}\sum_{n=0}^{5}\frac{r_{n}^{\prime }}{n!}{\left(\eta -\eta_{c}\right)}^{n}\right\}\exp\left\{-j\pi \frac{f_{\tau }^{2}}{K_{r}}\right\}$$

$r_{n}^{\prime }$ represents the modified *n*th-order slant range coefficient, which is:

(33) $$\left\{ \begin{array}{l} r_{0}^{\prime }\approx r_{0,ref}+\Delta R\\ r_{1}^{\prime }\approx -\frac{{k_{2,1,a}|}_{\Delta R=0}}{2}\eta_{c}^{2}\\ r_{2}^{\prime }\approx r_{2,ref}+k_{2,1,r}\cdot \Delta R+k_{2,2,r}\cdot {\left(\Delta R\right)}^{2}+\left[k_{2,2,a}-\frac{{k_{3,1,a}|}_{\Delta R=0}}{2}\right]\cdot {\left(\eta_{c}\right)}^{2}\\ r_{3}^{\prime }\approx r_{3,ref}-{k_{2,1,a}|}_{\Delta R=0}+k_{3,1,r}\cdot \Delta R+\left[k_{3,2,a}-\frac{{k_{4,1,a}|}_{\Delta R=0}}{2}\right]\cdot {\left(\eta_{c}\right)}^{2}\\ r_{4}^{\prime }\approx r_{4,ref}-{k_{3,1,a}|}_{\Delta R=0}+k_{4,1,r}\cdot \Delta R\\ r_{5}^{\prime }\approx r_{5,ref}-{k_{4,1,a}|}_{\Delta R=0} \end{array}\right.$$

By performing the azimuth FT on Equation (32), the signal in the two-dimensional frequency domain is:

(34) $$S_{2df}^{\prime }\left(f_{\eta },f_{\tau }\right)=\sigma \cdot W_{a}\left[f_{\eta }\right]\cdot rect\left[\frac{f_{\tau }}{B_{r}}\right]\exp\left\{j2\pi \sum_{k=1}^{9}\phi_{k}^{\prime }f_{\tau }^{k}\right\}\exp\left\{j2\pi \varphi_{0}^{\prime }\right\}\exp\left\{-j2\pi f_{\eta }\eta_{c}\right\}$$

where $\varphi_{0}^{\prime }$ and $\phi_{k}^{\prime }$ (1≤k≤9) can be attained by replacing $r_{n}$ with $r_{n}^{\prime }$ in Equation (11) and polynomial fitting, i.e.,

(35) $$\left\{ \begin{array}{l} \varphi_{0}^{\prime }\approx \varphi_{0,ref}^{\prime }+M_{1,r}^{\prime }\Delta R+M_{2,r}^{\prime }{\left(\Delta R\right)}^{2}+M_{2,a}^{\prime }\eta_{c}^{2}\\ \phi_{1}^{\prime }\approx \phi_{1,ref}^{\prime }-2/c_{0}\cdot {r_{pt}|}_{\eta =\eta_{c}}+L_{1,r}^{\prime }\Delta R+L_{2,r}^{\prime }{\left(\Delta R\right)}^{2}+L_{2,a}^{\prime }\eta_{c}^{2}\\ \phi_{2}^{\prime }\approx \phi_{2,ref}^{\prime }+J_{1,r}^{\prime }\Delta R+J_{2,a}^{\prime }\eta_{c}^{2}\\ \phi_{3}^{\prime }\approx \phi_{3,ref}^{\prime }+K_{1,r}^{\prime }\Delta R\\ \phi_{k}^{\prime }\approx \phi_{k,ref}^{\prime },4\leq k\leq 9 \end{array}\right.$$

After first azimuth time scaling, linear azimuthal variance is removed from $\phi_{k}^{\prime }$ in Equation (35), and the residual quadratic azimuth variance makes RCM variation be less than one quarter of a range cell in azimuth. Therefore, RCMs corresponding to targets in the same range cell can be regarded as identical [22], indicating that the first azimuth time scaling is beneficial to RCMC and range compression in the next step.

However, for targets in the same range cell a range offset in the RD domain is induced by the first azimuth scaling, which is:

(36) $$\frac{2{r_{pt}|}_{\eta =\eta_{c}}}{c_{0}}=\left(-\phi_{1}^{\prime }\right)-\left(-{\phi_{1}^{\prime }|}_{\eta_{c}=0}\right)=-\sum_{m=2}^{4}\frac{2{k_{m,1,a}|}_{\Delta R=0}}{c_{0}\left(m+1\right)!}\eta_{c}^{m+1}$$

**Figure 3:**
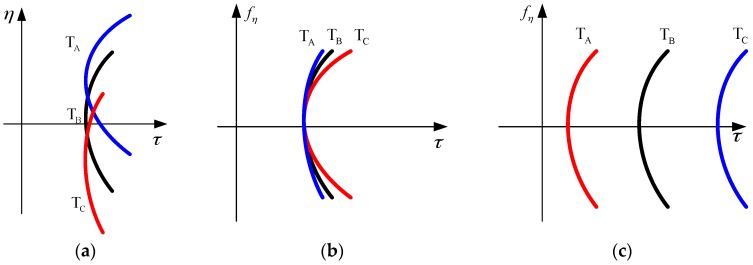
Illustration of first azimuth time scaling. Three targets (T_A_, T_B_ and T_C_) are in the same range cell. Because of azimuthal variance, their RCM curves vary in the time domain, as shown in (**a**); In the RD domain, only some parts of the curves coincide, as shown in (**b**); After first azimuth scaling, linear azimuth variance is corrected and the three curves in the RD domain are parallel, as demonstrated in (**c**).

In Figure 3a, three curves show RCMs corresponding to the three targets T_A_, T_B_ and T_C_. And, they are in the same range cell. These curves do not coincide because of azimuthal variance in the RD domain, as demonstrated in Figure 3b. After the first azimuth time scaling, RCMs are corrected to be the same and shifted by various offsets. As a result, they are approximately parallel in the RD domain and cross different range cells, as shown in Figure 3c. The range offset causes imaging distortion and is corrected in the final step.

### 4.2. RCM Correction and Range Compression

In order to correct the range variance of $\phi_{1}^{\prime }$, $\phi_{2}^{\prime }$, and $\phi_{3}^{\prime }$, the following compensation function can be applied based on the thought of time-frequency scaling which is demonstrated in Section 3:

(37) $$H_{4plus\_Y}\left(f_{\eta },f_{\tau }\right)=\exp\left\{-j2\pi \sum_{k=4}^{9}\phi_{k,ref}^{\prime }f_{\tau }^{k}\right\}\exp\left\{j\frac{2\pi }{3}Yf_{\tau }^{3}\right\}$$

where:

$$Y=\frac{2\beta K_{mref}+(1+2\alpha )K_{s}}{2\alpha (1+\alpha )K_{mref}^{3}}-3\phi_{3,ref}^{\prime }$$

$$\alpha =\frac{L_{1,r}^{\prime }}{L_{1,r,ref}^{\prime }}-1$$

$$\beta =\frac{L_{1,r}^{\prime }L_{2,r,ref}^{\prime }-L_{2,r}^{\prime }L_{1,r,ref}^{\prime }}{{\left(L_{1,r,ref}^{\prime }\right)}^{3}}$$

$$K_{mref}=\frac{1}{2\phi_{2,ref}^{\prime }}$$

$$K_{s}=\frac{2K_{mref}^{2}J_{1,r}^{\prime }}{L_{1,r,ref}^{\prime }}$$

$$L_{1,r,ref}^{\prime }={L_{1,r}^{\prime }|}_{f_{\eta }=f_{\eta ref}}$$

$$L_{2,r,ref}^{\prime }={L_{2,r}^{\prime }|}_{f_{\eta }=f_{\eta ref}}$$

and $f_{\eta ref}$ is the Doppler centroid corresponding to the swath center.

By applying range IFT on the multiplication of Equations (34) and (37), the signal in the RD domain can be formulated as follows (see Appendix C):

(38) $$\begin{array}{ll} S_{rd}\left(f_{\eta },\tau_{1}\right) & =\sigma \cdot W_{a}\left[f_{\eta }\right]\cdot rect\left[\frac{\tau_{1}-t_{con}-\alpha \tau^{\prime }-\beta {\left(\tau^{\prime }\right)}^{2}}{T_{p}}\right]\cdot \exp\left\{j2\pi \varphi_{0}^{\prime }\right\}\cdot \exp\left\{-j2\pi f_{\eta }\eta_{c}\right\}\\ & \cdot \exp\left\{-j\pi \left(K_{mref}+K_{s}\tau^{\prime }\right){\left[\tau_{1}-t_{con}-\alpha \tau^{\prime }-\beta {\left(\tau^{\prime }\right)}^{2}\right]}^{2}\right\}\\ & \cdot \exp\left\{-j\frac{2\pi }{3}\left(J_{mref}+J_{s}\tau^{\prime }\right){\left[\tau_{1}-t_{con}-\alpha \tau^{\prime }-\beta {\left(\tau^{\prime }\right)}^{2}\right]}^{3}\right\} \end{array}$$

where:

$$\tau^{\prime }=-L_{1,r,ref}^{\prime }\Delta R-L_{2,r,ref}^{\prime }{\left(\Delta R\right)}^{2}$$

$$\tau_{1}=\tau -\left(-\phi_{1,ref}^{\prime }-{\phi_{1}^{\prime }|}_{f_{\eta }=f_{\eta ref}}+{\phi_{1,ref}^{\prime }|}_{f_{\eta }=f_{\eta ref}}\right)$$

$$t_{con}=\frac{2{r_{pt}|}_{\eta =\eta_{c}}}{c_{0}}$$

$$J_{mref}=\left(3\phi_{3,ref}^{\prime }+Y\right)K_{mref}^{3}$$

$$J_{s}=-\frac{3K_{1,r}^{\prime }K_{mref}^{3}}{L_{1,r,ref}^{\prime }}+\frac{6J_{1,r}^{\prime }K_{mref}J_{mref}}{L_{1,r,ref}^{\prime }}$$

Coupling of $\tau_{1}$ and $\tau^{\prime }$ denotes range variance. Per Equation (24), the function for correcting the range variance is:

(39) $$H_{NCS}\left(f_{\eta },\tau_{1}\right)=\exp\left[-j\pi Q_{2}{\left(\tau_{1}+\tau^{\prime }\right)}^{2}-j\frac{2\pi }{3}Q_{3}{\left(\tau_{1}+\tau^{\prime }\right)}^{3}\right]$$

where $\tau_{1}+\tau^{\prime }=\tau +\phi_{1,ref}^{\prime }$, and:

(40) $$\{\begin{array}{l} Q_{2}=\alpha K_{mref}\\ Q_{3}=\frac{\beta K_{mref}+0.5\alpha K_{s}}{1+\alpha } \end{array}$$

The multiplication of Equations (38) and (39) can be approximated as:

(41) $$\begin{array}{ll} S_{rd}^{\prime \prime }\left(f_{\eta },\tau_{1}\right) & \approx \sigma \cdot W_{a}\left[f_{\eta }\right]\cdot rect\left[\frac{\tau_{1}-t_{con}-\alpha \cdot \tau^{\prime }-\beta \cdot {\left(\tau^{\prime }\right)}^{2}}{T_{p}}\right]\cdot \exp\left\{j2\pi \varphi_{0}^{\prime }\right\}\cdot \exp\left\{-j2\pi f_{\eta }\eta_{c}\right\}\\ & \cdot \exp\left\{-j\frac{2\pi }{3}\left(J_{mref}+Q_{3}\right)\cdot {\left(\tau_{1}\right)}^{3}\right\}\cdot \exp\left\{-j\pi \left(K_{mref}+Q_{2}\right){\left(\tau_{1}\right)}^{2}\right\}\\ & \cdot \exp\left\{-j\pi \left(K_{mref}+K_{s}\tau^{\prime }\right){\left[\alpha \tau^{\prime }+\beta {\left(\tau^{\prime }\right)}^{2}\right]}^{2}\right\}\cdot \exp\left\{j\frac{2\pi }{3}\left(J_{mref}+J_{s}\tau^{\prime }\right){\left[\alpha \tau^{\prime }+\beta {\left(\tau^{\prime }\right)}^{2}\right]}^{3}\right\}\\ & \cdot \exp\left\{-j\pi Q_{2}{\left(\tau^{\prime }\right)}^{2}-j\frac{2\pi }{3}Q_{3}{\left(\tau^{\prime }\right)}^{3}\right\}\cdot \exp\left[j2\pi \frac{K_{1,r}^{\prime }K_{mref}^{3}}{L_{1,r,ref}^{\prime }}\tau^{\prime }{\left(\tau_{1}\right)}^{3}\right] \end{array}$$

The last term of Equation (41) will result in asymmetric range sidelobe. To further eliminate the impact of range variance in the last term of Equation (41), data segmentation can be adopted in the RD domain. Every segment corresponds to a sub-swath whose width in the slant range direction is <30 km. The phase variation induced by the term with $\tau^{\prime }{\left(\tau_{1}\right)}^{3}$ is <0.04π in the sub-swath, which indicates that the last term in Equation (41) can be ignored in the processing of every segment.

By applying the range FT and the compensation function to Equation (41) successively, the range variance in every segment can be corrected. The compensation function is:

(42) $$H_{3\_sub}\left(f_{\eta },f_{\tau }\right)=\exp\left\{-j2\pi \frac{K_{mref}^{3}K_{1,r}^{\prime }\Delta R_{sub}}{{\left(K_{mref}+Q_{2}\right)}^{3}}f_{\tau }^{3}\right\}$$

where $\Delta R_{sub}$ is distance from the swath center to sub-swath center in the slant range direction. Then, compensation results of data segments are stitched together. A uniform filter can be applied to accomplish RCM correction and range compression for the entire swath, which is:

(43) $$\begin{array}{ll} H_{rpc\_rcmc}(f_{\eta },f_{\tau }) & =\exp\left\{-j\left[\frac{\pi f_{\tau }^{2}}{K_{mref}+Q_{2}}-\frac{2\pi }{3}\frac{J_{mref}+Q_{3}}{{\left(K_{mref}+Q_{2}\right)}^{3}}f_{\tau }^{3}\right]\right\}\\ & \cdot \exp\left\{j2\pi \left(\tau_{mig,ref}^{\prime }-{\tau_{mig,ref}^{\prime }|}_{f_{\eta }=f_{\eta ref}}\right)f_{\tau }\right\} \end{array}$$

By applying Equation (43), the spectrum in the RD domain becomes:

(44) $$\begin{array}{ll} S_{rd}^{\prime \prime \prime }\left(f_{\eta },\tau^{\prime }\right) & =\sigma \cdot W_{a}\left[f_{\eta }\right]\cdot sinc\left[\tau -{\tau_{mig}^{\prime }|}_{f_{\eta }=f_{\eta ref}}\right]\exp\left\{j2\pi \varphi_{0}^{\prime }\right\}\exp\left\{-j2\pi f_{\eta }\eta_{c}\right\}\\ & \cdot \exp\left\{-j\pi \left(K_{mref}+K_{s}\tau^{\prime }\right){\left[\alpha \tau^{\prime }+\beta {\left(\tau^{\prime }\right)}^{2}\right]}^{2}\right\}\\ & \cdot \exp\left\{j\frac{2\pi }{3}\left(J_{mref}+J_{s}\tau^{\prime }\right){\left[\alpha \tau^{\prime }+\beta {\left(\tau^{\prime }\right)}^{2}\right]}^{3}\right\}\\ & \cdot \exp\left\{-j\pi Q_{2}{\left(\tau^{\prime }\right)}^{2}-j\frac{2\pi }{3}Q_{3}{\left(\tau^{\prime }\right)}^{3}\right\} \end{array}$$

The last three terms in Equation (44) can be further compensated for by the following function:

(45) $$\begin{array}{ll} H_{left}\left(f_{\eta },\tau \right) & =\exp\left\{-j\pi \left[K_{mref}+K_{s}\cdot \left(\tau -\frac{2r_{0,ref}}{c_{0}}\right)\right]{\left[\alpha \cdot \left(\tau -\frac{2r_{0,ref}}{c_{0}}\right)+\beta \cdot {\left(\tau -\frac{2r_{0,ref}}{c_{0}}\right)}^{2}\right]}^{2}\right\}\\ & \exp\left\{j\frac{2\pi }{3}\left[J_{mref}+J_{s}\cdot \left(\tau -\frac{2r_{0,ref}}{c_{0}}\right)\right]{\left[\alpha \cdot \left(\tau -\frac{2r_{0,ref}}{c_{0}}\right)+\beta \cdot {\left(\tau -\frac{2r_{0,ref}}{c_{0}}\right)}^{2}\right]}^{3}\right\}\\ & \exp\left\{-j\pi Q_{2}\cdot {\left(\tau -\frac{2r_{0,ref}}{c_{0}}\right)}^{2}-j\frac{2\pi }{3}Q_{3}\cdot {\left(\tau -\frac{2r_{0,ref}}{c_{0}}\right)}^{3}\right\} \end{array}$$

Compensated for by Equation (45), the signal is:

(46) $$S_{rd}^{\left(4\right)}\left(f_{\eta },\tau \right)=\sigma \cdot W_{a}\left[f_{\eta }\right]\cdot sinc\left[\tau -{\tau_{mig}^{\prime }|}_{f_{\eta }=f_{\eta ref}}\right]\exp\left\{j2\pi \varphi_{0}^{\prime }\right\}\exp\left\{-j2\pi f_{\eta }\eta_{c}\right\}$$

### 4.3. Second Azimuth Time-Frequency Scaling

After RCM correction and range compression, the third step is to further correct the residual azimuth variance in $\varphi_{0}^{\prime }$.

To compensate for the additional phase induced by the first azimuth time scaling, the following function is adopted, which is:

(47) $$H_{-AS1}\left(\eta ,\tau \right)=\exp\left\{j\frac{4\pi }{\lambda }r_{pt}\left(\eta \right)\right\}$$

By applying the azimuth IFT and Equation (47) to Equation (46) successively, the signal in the RD domain is:

(48) $$\begin{array}{ll} S_{rd}^{\left(5\right)}\left(f_{\eta },\tau \right) & =\sigma \cdot W_{a}\left[f_{\eta }\right]\cdot sinc\left[\tau -{{\tau^{\prime }}_{mig}|}_{f_{\eta }=f_{\eta ref}}\right]\cdot \exp\left\{-j2\pi f_{\eta }\eta_{c}\right\}\\ & \cdot \exp\left\{j2\pi \left[-\frac{2}{\lambda }\left(r_{0}-P_{0}\right)+\sum_{m=1}^{10}\frac{2P_{m}}{\lambda }{\left(\frac{\lambda }{2}f_{\eta }\right)}^{m}\right]\right\} \end{array}$$

where the last phase is spatially variant and leads to azimuth defocusing.

Based on the basic methodology in Section 3, to correct the azimuthal variance in Equation (48), the frequency scaling is adopted, which is:

(49) $$H_{Y_{3}Y_{4}}\left(f_{\eta }\right)=\exp\left\{j\frac{2\pi Y_{3}}{3\lambda }{\left(\frac{\lambda }{2}\right)}^{3}f_{\eta }^{3}-j\frac{\pi Y_{4}}{\lambda }{\left(\frac{\lambda }{2}\right)}^{4}f_{\eta }^{4}\right\}$$

where:

(50) $$Y_{3}=\frac{1}{2k_{2,1,a}\cdot {\left({r_{2}|}_{\eta_{c}=0}\right)}^{3}}\left[-k_{2,1,a}^{2}+{r_{3}|}_{\eta_{c}=0}\cdot k_{2,1,a}+\left(2k_{2,2,a}-k_{3,1,a}\right)\cdot {r_{2}|}_{\eta_{c}=0}\right]$$

and:

(51) $$\begin{array}{ll} Y_{4} & =-\frac{1}{36k_{2,1,a}^{2}{\left({r_{2}|}_{\eta_{c}=0}\right)}^{5}}[12k_{2,1,a}^{2}\cdot {\left({r_{3}|}_{\eta_{c}=0}\right)}^{2}+12k_{2,2,a}^{2}\cdot {\left({r_{2}|}_{\eta_{c}=0}\right)}^{2}-21k_{2,1,a}^{3}\cdot {r_{3}|}_{\eta_{c}=0}+9k_{2,1,a}^{4}\\ & -24k_{2,1,a}^{2}\cdot k_{2,2,a}\cdot {r_{2}|}_{\eta_{c}=0}+11k_{2,1,a}^{2}\cdot k_{3,1,a}\cdot {r_{2}|}_{\eta_{c}=0}-4k_{2,1,a}\cdot k_{3,2,a}\cdot {\left({r_{2}|}_{\eta_{c}=0}\right)}^{2}\\ & -6k_{2,2,a}\cdot k_{3,1,a}\cdot {\left({r_{2}|}_{\eta_{c}=0}\right)}^{2}+2k_{2,1,a}\cdot k_{4,1,a}\cdot {\left({r_{2}|}_{\eta_{c}=0}\right)}^{2}-2k_{2,1,a}^{2}\cdot {r_{2}|}_{\eta_{c}=0}\cdot {r_{4}|}_{\eta_{c}=0}\\ & +30k_{2,1,a}\cdot k_{2,2,a}\cdot {r_{2}|}_{\eta_{c}=0}\cdot {r_{3}|}_{\eta_{c}=0}-12k_{2,1,a}\cdot k_{3,1,a}\cdot {r_{2}|}_{\eta_{c}=0}\cdot {r_{3}|}_{\eta_{c}=0}] \end{array}$$

After multiplying Equation (48) with Equation (49), the signal in the time domain is:

(52) $$s_{2dt}^{\prime \prime }\left(\eta ,\tau \right)=\sigma \cdot rect\left[\frac{\eta -\eta_{c}}{T_{s}}\right]\cdot sinc\left[\tau -{\tau_{mig}^{\prime }|}_{f_{\eta }=f_{\eta ref}}\right]\exp\left\{-j\frac{4\pi }{\lambda }\sum_{n=0}^{5}\frac{r_{n}^{\prime \prime }}{n!}{\left(\eta -\eta_{c}\right)}^{n}\right\}$$

where:

(53) $$\left\{ \begin{array}{l} r_{0}^{\prime \prime }=r_{0}\\ r_{1}^{\prime \prime }=r_{1}\\ r_{2}^{\prime \prime }=r_{2}\\ r_{3}^{\prime \prime }=r_{3}+Y_{3}r_{2}^{3}\\ r_{4}^{\prime \prime }=r_{4}+3Y_{3}^{2}r_{2}^{5}+6r_{3}Y_{3}r_{2}^{2}+6Y_{4}r_{2}^{4}\\ r_{5}^{\prime \prime }=r_{5}+15Y_{3}^{3}r_{2}^{7}+45Y_{3}^{2}r_{2}^{4}r_{3}+60Y_{3}Y_{4}r_{2}^{6}+10Y_{3}r_{2}^{2}r_{4}+15Y_{3}r_{2}r_{3}^{2}+60Y_{4}r_{2}^{3}r_{3} \end{array}\right.$$

Then, the azimuth time scaling function is applied to Equation (52), which is:

(54) $$H_{AS2}\left(\eta \right)=\exp\left\{-j\frac{4\pi }{\lambda }r_{pt}^{\prime }\left(\eta \right)\right\}$$

where:

(55) $$r_{pt}^{\prime }\left(\eta \right)=\sum_{n=3}^{5}\frac{p_{n}}{n!}\eta^{n}$$

and:

(56) $$\{\begin{array}{l} p_{3}=-k_{2,1,a}\\ p_{4}=-k_{3,1,a}-3Y_{3}\cdot k_{2,1,a}\cdot {\left({r_{2}|}_{\eta_{c}=0}\right)}^{2}\\ p_{5}=-k_{4,1,a}-15Y_{3}^{2}\cdot {\left({r_{2}|}_{\eta_{c}=0}\right)}^{4}-6k_{3,1,a}\cdot Y_{3}\cdot {\left({r_{2}|}_{\eta_{c}=0}\right)}^{2}-12Y_{3}\cdot k_{2,1,a}\cdot {r_{2}|}_{\eta_{c}=0}\cdot {r_{3}|}_{\eta_{c}=0}-24Y_{4}\cdot k_{2,1,a}\cdot {\left({r_{2}|}_{\eta_{c}=0}\right)}^{3} \end{array}$$

After compensated for by Equation (54), Equation (52) becomes:

(57) $$s_{2dt}^{\prime \prime \prime }\left(\eta ,\tau \right)=\sigma \cdot rect\left[\frac{\eta -\eta_{c}}{T_{s}}\right]\cdot sinc\left[\tau -{\tau_{mig}^{\prime }|}_{f_{\eta }=f_{\eta ref}}\right]\exp\left\{-j\frac{4\pi }{\lambda }\sum_{n=0}^{5}\frac{r_{n}^{\prime \prime \prime }}{n!}{\left(\eta -\eta_{c}\right)}^{n}\right\}$$

where:

(58) $$\{\begin{array}{l} r_{n}^{\prime \prime \prime }=r_{n}^{\prime \prime }+\sum_{m=3}^{5}\frac{p_{m}}{\left(m-n\right)!}{\eta_{c}}^{m-n},0\leq n\leq 2\\ r_{n}^{\prime \prime \prime }=r_{n}^{\prime \prime }+\sum_{m=n}^{5}\frac{p_{m}}{(m-n)!}{t_{c}}^{m-n},3\leq n\leq 5 \end{array}$$

By applying azimuth FT to Equation (57), the signal becomes:

(59) $$\begin{array}{ll} S_{rd}^{\left(7\right)}\left(f_{\eta },\tau \right) & =\sigma \cdot W_{a}\left[f_{\eta }\right]\cdot sinc\left[\tau -{\tau_{mig}^{\prime }|}_{f_{\eta }=f_{\eta ref}}\right]\exp\left\{-j\frac{4\pi }{\lambda }\left(r_{0}^{\prime \prime \prime }-P_{0}^{\prime \prime \prime }\right)-j2\pi f_{\eta }\left(\eta_{c}-P_{1}^{\prime \prime \prime }\right)\right\}\\ & \cdot \exp\left\{j\sum_{m=2}^{10}\frac{4\pi P_{m}^{\prime \prime \prime }}{\lambda }{\left(\frac{\lambda }{2}f_{\eta }\right)}^{m}\right\} \end{array}$$

where $P_{m}^{\prime \prime \prime }$ can be obtained by replacing $r_{n}$ with $r_{n}^{\prime \prime \prime }$ in Equation (12). The spectrum envelope can be expressed as:

(60) $$\begin{array}{ll} W_{a}\left[f_{\eta }\right] & \approx 1-\frac{r_{3}^{\prime \prime \prime }}{2{\left(r_{2}^{\prime \prime \prime }\right)}^{2}}\left(-\frac{\lambda }{2}f_{\eta }\right)-\frac{2r_{2}^{\prime \prime \prime }\cdot r_{4}^{\prime \prime \prime }-5{\left(r_{3}^{\prime \prime \prime }\right)}^{2}}{8{\left(r_{2}^{\prime \prime \prime }\right)}^{4}}{\left(-\frac{\lambda }{2}f_{\eta }\right)}^{2}\\ & -\frac{4r_{5}^{\prime \prime \prime }\cdot {\left(r_{2}^{\prime \prime \prime }\right)}^{2}-34r_{2}^{\prime \prime \prime }\cdot r_{3}^{\prime \prime \prime }\cdot r_{4}^{\prime \prime \prime }+45{\left(r_{3}^{\prime \prime \prime }\right)}^{3}}{48{\left(r_{2}^{\prime \prime \prime }\right)}^{6}}{\left(-\frac{\lambda }{2}f_{\eta }\right)}^{3} \end{array}$$

In Equation (59), the phase variation induced by azimuthal variance is <0.024π in the entire swath, and does not affect azimuth focusing. As a result, the azimuth focusing can be implemented in the frequency domain.

### 4.4. Azimuth Compression

Equation (59) is spatially invariant and can be focused by applying a uniform azimuth matching filter for the entire swath, i.e.,

(61) $$H_{apc}(f_{\eta })=\frac{1}{A_{amp}(f_{\eta })}\exp\left\{-j2\pi \varphi_{r_{c},0}^{\prime \prime }\right\}$$

where:

(62) $$\{\begin{array}{l} \varphi_{r_{c},0}^{\prime \prime }=\sum_{m=2}^{10}\frac{2P_{r_{c},m}}{\lambda }{\left(\frac{\lambda }{2}f_{\eta }\right)}^{m}\\ \begin{array}{ll} A_{amp}(f_{\eta }) & =1-\frac{r_{c,3}}{2r_{c,2}^{2}}\left(-\frac{\lambda }{2}f_{\eta }\right)-\frac{2r_{c,2}\cdot r_{c,4}-5r_{c,3}^{2}}{8r_{c,2}^{4}}{\left(-\frac{\lambda }{2}f_{\eta }\right)}^{2}\\ & -\frac{4r_{c,5}\cdot r_{c,2}^{2}-34r_{c,2}\cdot r_{c,3}+30r_{c,3}^{3}}{48r_{c,2}^{6}}{\left(-\frac{\lambda }{2}f_{\eta }\right)}^{3} \end{array} \end{array}$$

and:

(63) $$\left\{ \begin{array}{ll} r_{c,0} & ={r_{0}|}_{\eta_{c}=0}\\ r_{c,1} & ={r_{1}|}_{\eta_{c}=0}\\ r_{c,2} & ={r_{2}|}_{\eta_{c}=0}\\ r_{c,3} & ={r_{3}|}_{\eta_{c}=0}+Y_{3}\cdot {\left({r_{2}|}_{\eta_{c}=0}\right)}^{3}+p_{3}\\ r_{c,4} & ={r_{4}|}_{\eta_{c}=0}+3Y_{3}^{2}\cdot {\left({r_{2}|}_{\eta_{c}=0}\right)}^{5}+6Y_{3}\cdot {r_{3}|}_{\eta_{c}=0}\cdot {\left({r_{2}|}_{\eta_{c}=0}\right)}^{2}+6Y_{4}\cdot {\left({r_{2}|}_{\eta_{c}=0}\right)}^{4}+p_{4}\\ r_{c,5} & ={r_{5}|}_{\eta_{c}=0}+15Y_{3}^{3}\cdot {\left({r_{2}|}_{\eta_{c}=0}\right)}^{7}+45Y_{3}^{2}\cdot {\left({r_{2}|}_{\eta_{c}=0}\right)}^{4}{\cdot r_{3}|}_{\eta_{c}=0}+60Y_{3}Y_{4}\cdot {\left({r_{2}|}_{\eta_{c}=0}\right)}^{6}\\ & +10Y_{3}\cdot {\left({r_{2}|}_{\eta_{c}=0}\right)}^{2}\cdot {r_{4}|}_{\eta_{c}=0}+15Y_{3}\cdot {r_{2}|}_{\eta_{c}=0}\cdot {\left({r_{3}|}_{\eta_{c}=0}\right)}^{2}+60Y_{4}\cdot {\left({r_{2}|}_{\eta_{c}=0}\right)}^{3}{\cdot r_{3}|}_{\eta_{c}=0}+p_{5} \end{array}\right.$$

$P_{r_{c},m}$ can be attained by replacing $r_{n}$ with $r_{c,n}$ in Equation (12). The result of matching filtering is:

(64) $$\begin{array}{ll} S_{rd}^{\left(8\right)}\left(f_{\eta },\tau \right) & \approx sinc\left[\tau -{{\tau^{\prime }}_{mig}|}_{f_{\eta }=f_{\eta ref}}\right]rect\left[\frac{f_{\eta }}{B_{a}}\right]\\ & \cdot \exp\left\{-j\frac{4\pi }{\lambda }\left(r_{0}^{\prime \prime \prime }-P_{0}^{\prime \prime \prime }\right)-j2\pi f_{\eta }\left(\eta_{c}-P_{1}^{\prime \prime \prime }\right)\right\} \end{array}$$

By implementing azimuth IFT, the focusing result can be expressed as:

(65) $$s_{2dt}^{\left(4\right)}\left(\eta ,\tau \right)=sinc\left[\tau -{\tau_{mig}^{\prime }|}_{\eta_{c}=0,f_{\eta }=f_{\eta ref}}-t_{con}\right]sinc\left[\eta -\left(\eta_{c}-P_{1}^{\prime \prime \prime }\right)\right]\cdot \exp\left\{-j\frac{4\pi }{\lambda }\left(r_{0}^{\prime \prime \prime }-P_{0}^{\prime \prime \prime }\right)\right\}$$

### 4.5. Geometric Correction

**Figure 4:**
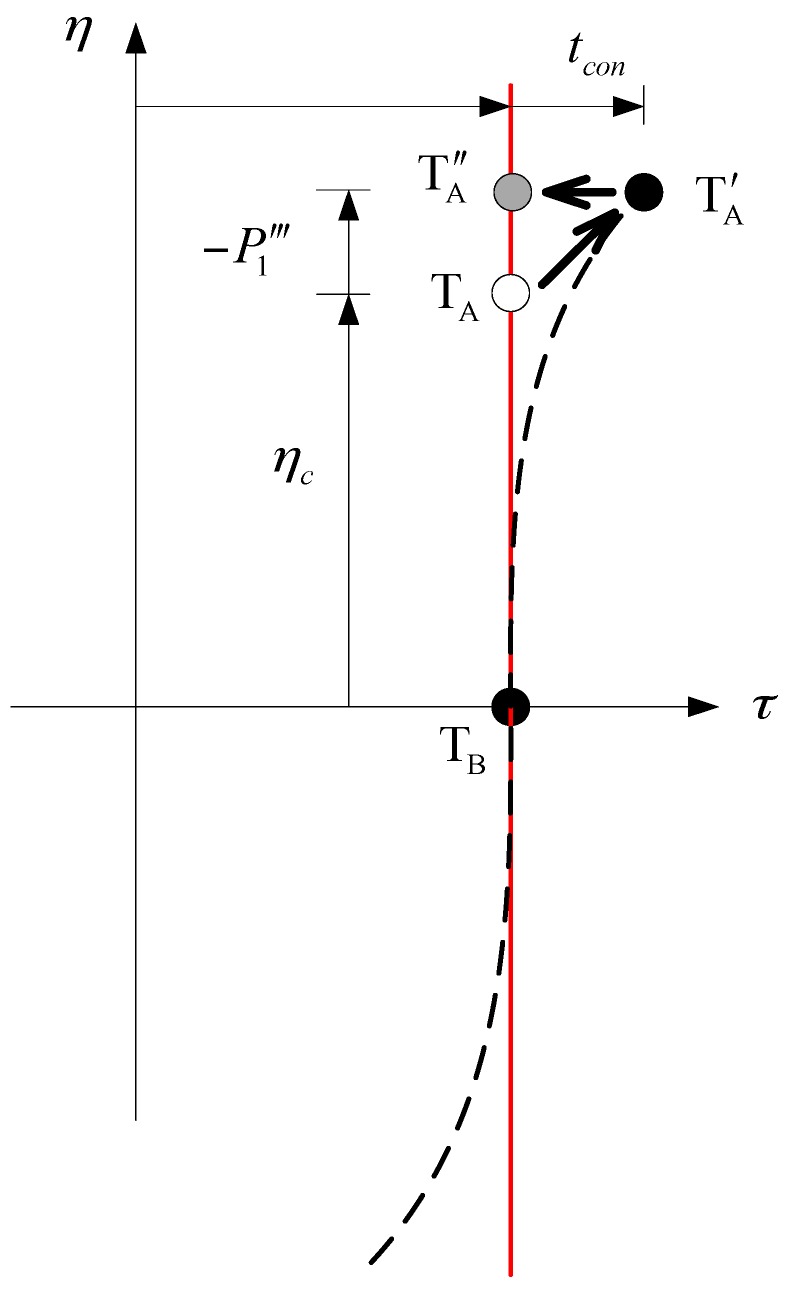
Illustration of geometric distortion. Two targets (T_A_ and T_B_) are orignally located in the same range cell, and $\eta_{c}$ for T_B_ is zero. After imaging by proposed algorithm, T_B_ is still at its original location. However, T_A_ is focused at $T_{A}^{\prime }$. Offsets in the range and azimuth directions are $t_{con}$ and $-P_{1}^{\prime \prime \prime }$ , respectively. All targets originally on red line are on dashed line after focusing. Therefore, the focusing results must be corrected from the dashed to red line by geometric correction, from $T_{A}^{\prime }$ to $T_{A}^{\prime \prime }$ .

Equation (65) indicates that the echo has been completely focused. However, the target location is shifted in both azimuth and range directions. As illustrated in Figure 4, two targets, T_A_ and T_B_, are originally in the same range cell, and $\eta_{c}$ for T_B_ is zero. In the focusing result, T_B_ is still at its original position. However, T_A_ has been shifted to another position $T_{A}^{\prime }$. The offsets along the range and azimuth directions are $t_{con}$ and $-P_{1}^{\prime \prime \prime }$, respectively, revealing geometric distortion in the focused imaging that should be corrected.

In Figure 4, the dashed line represents the offset trajectory, which can be described by:

(66) $$g(\eta )=\frac{2}{c_{0}}\left[\frac{G_{3}}{6}\eta^{3}+\frac{G_{4}}{24}\eta^{4}+\frac{G_{5}}{120}\eta^{5}\right]$$

where:

(67) $$\{\begin{array}{l} G_{3}=p_{3}\\ G_{4}=p_{4}+12G_{3}\cdot Q_{1,2,a}\\ G_{5}=p_{5}+20G_{4}\cdot Q_{1,2,a}+60G_{3}\cdot \left(Q_{1,3,a}-Q_{1,2,a}^{2}\right) \end{array}$$

$Q_{1,2,a}$ and $Q_{1,3,a}$ are the second-order and the third-order spatial variance coefficients of $P_{1}^{\prime \prime \prime }$ along the azimuth, respectively. They can be obtained by replacing $r_{n}$ with $r_{n}^{\prime \prime \prime }$ in Equations (9) and (12).

Equation (65) can be transformed into the range-frequency and azimuth-time domain, multiplied by the geometric correction function, i.e.,

(68) $$H_{geoCorrect}\left(\eta ,f_{\tau }\right)=\exp\left[j2\pi f_{\tau }\cdot g\left(\eta \right)\right]$$

and transformed back into the time domain to achieve the final imaging result, which is:

(69) $$s_{2dt}^{\left(5\right)}\left(\eta ,\tau \right)=sinc\left[\tau -{\tau_{mig}^{\prime }|}_{\eta_{c}=0,f_{\eta }=f_{\eta ref}}\right]sinc\left[\eta -\left(\eta_{c}-P_{1}^{\prime \prime \prime }\right)\right]\cdot \exp\left\{-j\frac{4\pi }{\lambda }\left(r_{0}^{\prime \prime \prime }-P_{0}^{\prime \prime \prime }\right)\right\}$$

**Figure 5:**
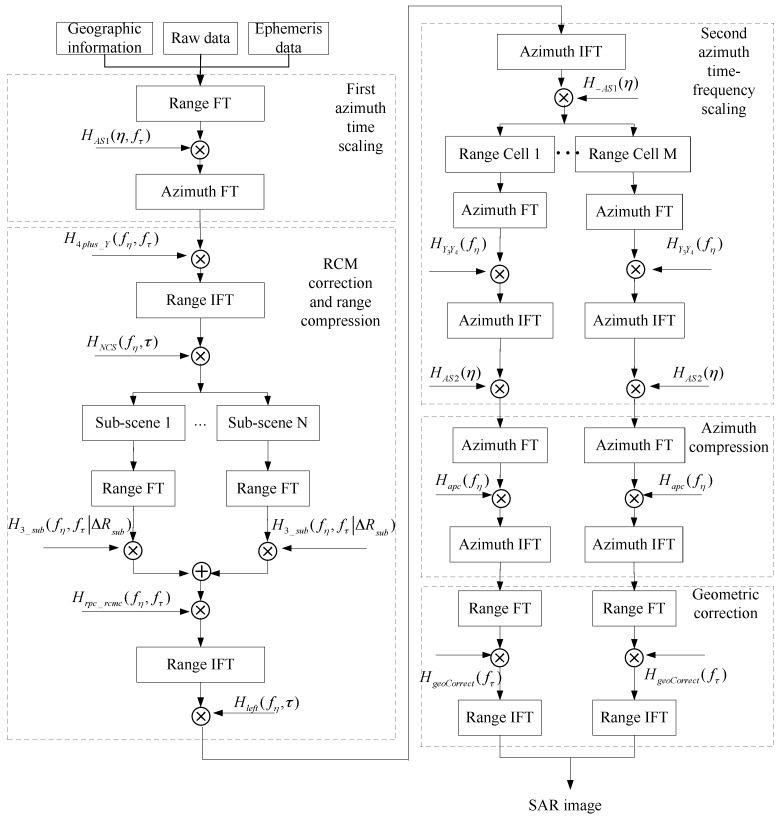
Flowchart of the proposed algorithm.

A flowchart of the proposed algorithm is shown in Figure 5.

## 5. Simulation and Verification

### 5.1. Simulation Parameters

Simulation parameters are listed in Table 2, which refer to the global earthquake satellite system [5]. The center of the swath is set to be 108.5° E and 35.3° N. Besides these parameters, the spatial variance of RCM also depends on the swath size. As the swath becomes wider, higher-order spatial variance may occur along both range and azimuth. As discussed in Section 4, the proposed algorithm can correct linear and quadric spatial variance. Therefore, in order to avoid the cubic and higher-order spatial variance, a simulated swath covering an area of 83 km (azimuth) × 86 km (range) is adopted to verify the algorithm with parameters in Table 2. It is certain that the swath size for the proposed algorithm varies with observation parameters. For example, if the center time is 0 and the swath center is at the equator, the swath size can reach 150 km (azimuth) × 150 km (range) (see Appendix D).

**Figure 6:**
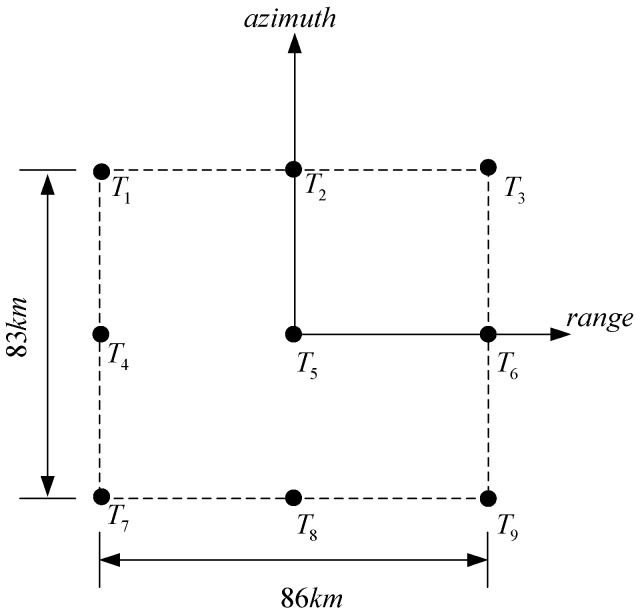
T_5_ is at swath center. T_1_ is 41.5 km and 43 km away from T_5_ along azimuth and range, respectively.

The simulated swath is portrayed in Figure 6. Nine point targets, from $T_{1}$ to $T_{9}$, are deployed. $T_{5}$ is at the swath center and other points are at the swath edge.

### 5.2. Imaging Results

**Figure 7:**
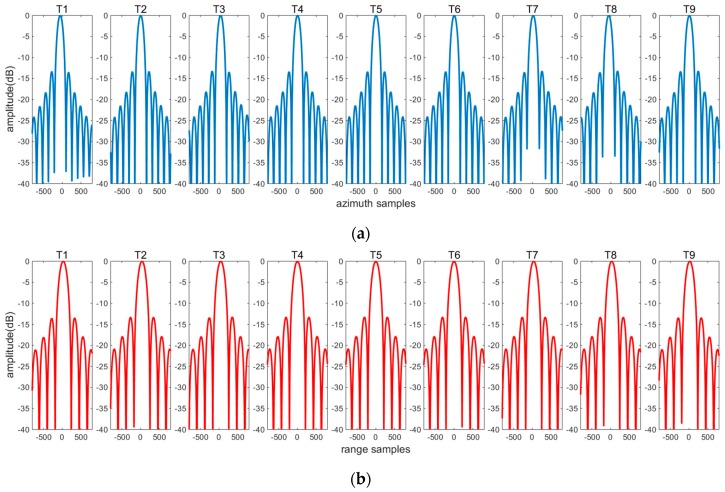
(**a**) Azimuth and (**b**) range profiles corresponding to every point target, represented by blue and red lines, respectively.

Range and azimuth profiles corresponding to every target are illustrated in Figure 7, and evaluation results are listed in Table 3. The broadening coefficient is the ratio of the achieved resolution to the ideal resolution. Suppose that $\rho_{a}$ and $\rho_{a,ideal}$ are the azimuth resolutions achieved by the proposed algorithm and the ideal imaging, respectively. The azimuth broadening coefficient is defined as $\rho_{a}/\rho_{a,ideal}$. The range broadening coefficient can be achieved by the same way. As shown in Table 3, the broadening coefficients are almost 1, which signifies no resolution loss in imaging. The ideal PSLR should be −13.26 dB. The loss of PSLR equals the absolute difference between the actual and ideal PSLRs. Table 3 shows that the maximal loss of range and azimuth PSLRs is ≤0.2 dB, and ≤0.1 dB, indicating good focusing quality. The difference of range PSLRs, azimuth PSLRs, range ISLRs, and azimuth ISLRs over the whole swath is ≤0.22 dB, ≤0.17 dB, ≤0.29 dB, and ≤0.28 dB respectively, indicating uniform imaging quality.

**Figure 8:**
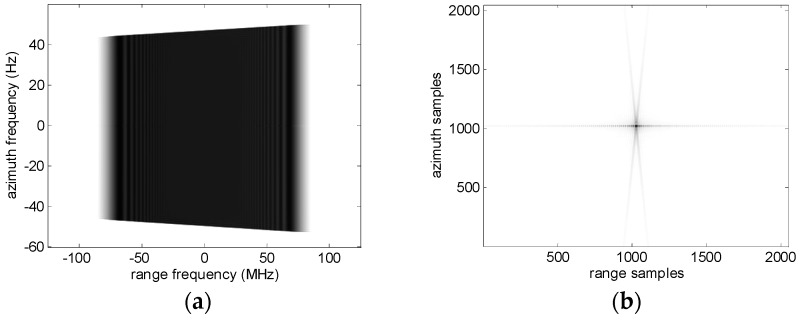
The amplitude spectrum (**a**) and the contour map (**b**) corresponding to the imaging result of T_5_.

Usually for LEO SAR azimuth ISLRs and range ISLRs are almost equal. The situation is different for GEO SAR with a resolution of 2 m. Because of the wide-angle observation, the two-dimensional amplitude spectrum is not rectangular, and bifurcation exists along azimuth, as shown in Figure 8. The bifurcation causes the energy of sidelobes to disperse in two directions, while the mainlobe is not affected. As a result, azimuth ISLRs are better than range ISLRs in Table 3.

In order to further demonstrate the advantage of the proposed algorithm, it is useful to compare the imaging results obtained by different techniques. Here the imaging results of T_3_ and T_5_ are compared by applying the proposed algorithm, and other two algorithms developed by Hu [15] and Li [17].

**Figure 9:**
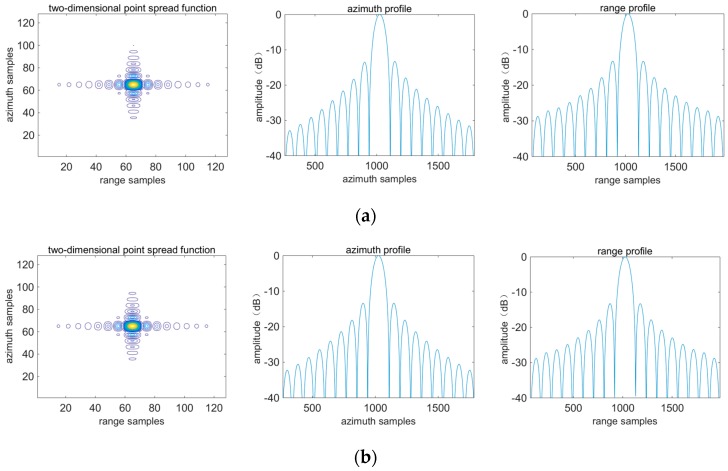

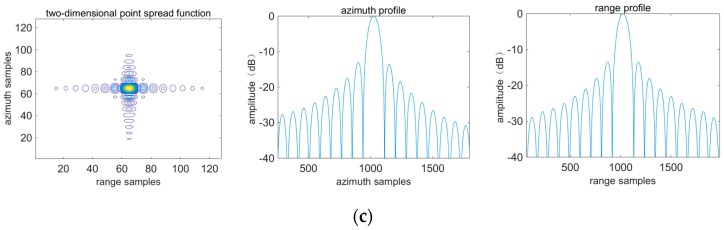
Imaging profiles corresponding to T_5_. The first column represents the two-dimensional point spread function. The second and third columns represent azimuth and range profiles. (**a**–**c**) are achieved by applying the proposed algorithm, the algorithm in [15] and the algorithm in [17] respectively.

**Figure 10:**
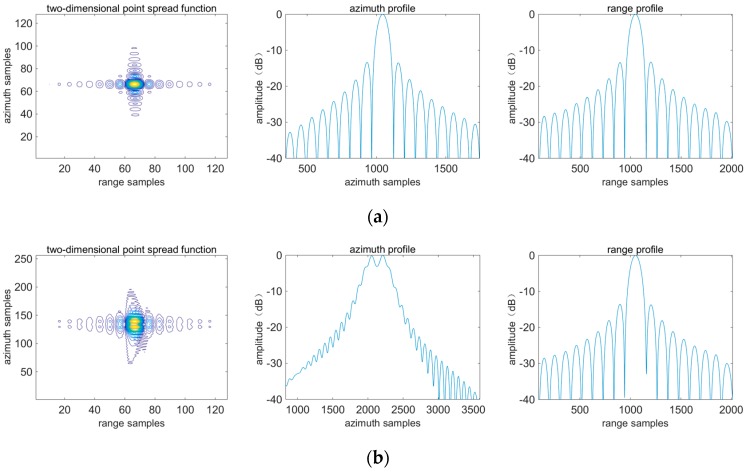

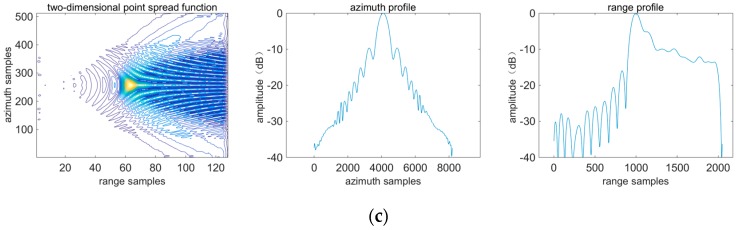
Imaging profiles corresponding to T_3_. The first column represents the two-dimensional point spread function. The second and third columns represent azimuth and range profiles. (**a**–**c**) are achieved by applying the proposed algorithm, the algorithm in [15] and the algorithm in [17] respectively.

Theoretically, any algorithm can achieve good focusing quality for T_5_, because it is at the swath center. For the same target at the swath edge the imaging performances of different algorithms may be different. Imaging profiles corresponding to T_5_ and T_3_ are shown in Figure 9 and Figure 10. Evaluation results are listed in Table 4 and Table 5. It is shown that for T_5_ three algorithms have basically the same imaging performance. However, for T_3_, azimuth defocusing occurs by applying the algorithm in [15]. The algorithm in [17] induces defocusing along azimuth and range directions, because the range variance is not corrected adequately and furthermore the azimuth focusing quality is affected. By comparison, the proposed algorithm has the best performance between these three algorithms.

### 5.3. Computational Load

Computational load is a key element to restrict the application of an algorithm. Although the chirp scaling algorithm (CSA) [12] cannot achieve the 2 m resolution for GEO SAR, it is worth comparing CSA and the proposed algorithm from the aspect of the computational load, because CSA is recognized as an efficient algorithm and has been widely applied. The back projection algorithm (BPA) [23] is also compared here, because BPA can achieve same focusing quality in the time domain.

Computational load is evaluated according to the complex multiplication and addition in the algorithm. Multiplication of two complex numbers and addition of two real numbers need 6 FLOPs and 1 FLOP, respectively. A FFT or IFFT with a length of *N* points needs $5N\log_{2}\left(N\right)$ FLOPs [18].

Suppose sampling numbers along azimuth and range are $N_{azi}$ and $N_{rng}$, respectively. The computational loads of the proposed algorithm, CSA, and BPA with 8-fold interpolation, are respectively:

(70) $$\left\{ \begin{array}{l} C_{P}=25N_{azi}N_{rng}\log_{2}\left(N_{rng}\right)+30N_{azi}N_{rng}\log_{2}\left(N_{azi}\right)+67N_{azi}N_{rng}\\ C_{CSA}=10N_{azi}N_{rng}\log_{2}\left(N_{rng}\right)+10N_{azi}N_{rng}\log_{2}\left(N_{azi}\right)+18N_{azi}N_{rng}\\ C_{BPA}=45N_{azi}N_{rng}\log_{2}\left(N_{rng}\right)+7N_{azi}^{2}N_{rng}+126N_{azi}N_{rng} \end{array}\right.$$

In order to achieve 2 m resolution and a swath of 80 km × 80 km, $N_{azi}$ and $N_{rng}$ should be 140,000 and 50,000 at least. According to Equation (70), analysis results are listed in Table 6. Although the computational load of the proposed algorithm is more than twice that of CSA, the increasement is acceptable because of the significant improvement of imaging quality. And the computational load is about 1/1000 of that of BPA, indicating that the proposed algorithm is efficient.

## 6. Conclusions

This work models the spatial variance in the time and frequency domains based on a fifth-order polynomial slant range model. And a GEO SAR imaging algorithm is proposed, whose basic method is to correct the linear and quadratic spatial variance of RCM in the range and azimuth directions based on time-frequency scaling. As demonstrated by simulation results, this algorithm can accomplish GEO SAR imaging with good and uniform imaging quality over the entire swath.

The algorithm can be applied in the squint mode for more flexible observation, although it is developed under the condition of zero Doppler centroid. For the squint mode, the Doppler centroid is spatially variant. As a result, data segmentation has to be used to divide the echo into blocks. Every block is processed by linear RCM correction [24] and the proposed algorithm successively. Imaging results of blocks are mosaicked to form the final image.

## Figures and Tables

**Table 1:** Analysis Parameters for Slant Range Order.

Parameters	Value
Orbital inclination angle	60°
Eccentricity	0
Wavelength	0.24 m
Incidence angle	35°
Ground resolution	2 m

**Table 2:** Parameters for simulation and verification.

Parameters	Value
Orbital inclination angle	60°
Eccentricity	0
Center time	8600 s
Wavelength	0.24 m
Pulse width	2 μs
Bandwidth	150 MHz
Sampling rate	250 MHz
Pulse repetition frequency	120 Hz
Incidence angle	35°
Squint angle	90°
Synthetic aperture time	750 s

**Table 3:** Evaluation Results.

Target	T_1_	T_2_	T_3_	T_4_	T_5_	T_6_	T_7_	T_8_	T_9_
Azimuth resolution (m)	1.87	1.86	1.88	1.91	1.91	1.91	1.96	1.96	1.97
Azimuth broadening	1.00	1.00	1.00	1.00	1.00	1.00	1.00	1.00	1.00
Azimuth PSLR (dB)	−13.29	−13.30	−13.23	−13.36	−13.36	−13.36	−13.22	−13.19	−13.24
Azimuth ISLR (dB)	−10.37	−10.51	−10.32	−10.60	−10.55	−10.55	−10.59	−10.54	−10.51
Range resolution (m)	1.95	1.95	1.94	1.94	1.94	1.94	1.94	1.94	1.94
Range broadening	1.00	1.00	1.00	1.00	1.00	1.00	1.00	1.00	1.00
Range PSLR (dB)	−13.46	−13.29	−13.24	−13.25	−13.26	−13.26	−13.26	−13.28	−13.40
Range ISLR (dB)	−9.98	−9.72	−9.76	−9.73	−9.69	−9.69	−9.74	−9.71	−9.84

**Table 4:** Evaluation Results Corresponding to T_5_.

	Proposed Algorithm	Algorithm in [15]	Algorithm in [17]
Azimuth resolution (m)	1.91	1.89	1.91
Azimuth broadening	1.00	1.00	1.00
Azimuth PSLR (dB)	−13.36	−13.37	−13.02
Azimuth ISLR (dB)	−10.55	−10.61	−10.25
Range resolution (m)	1.94	1.94	1.94
Range broadening	1.00	1.00	1.00
Range PSLR (dB)	−13.26	−13.25	−13.42
Range ISLR (dB)	−9.69	−9.68	−9.78

**Table 5:** Evaluation Results Corresponding to T_3_.

	Proposed Algorithm	Algorithm in [15]	Algorithm in [17]
Azimuth resolution (m)	1.88	3.71	15.48
Azimuth broadening	1.00	1.86	7.77
Azimuth PSLR (dB)	−13.23	−0.16	−9.63
Azimuth ISLR (dB)	−10.32	0.09	−7.42
Range resolution (m)	1.94	1.94	2.38
Range broadening	1.00	1.00	1.204
Range PSLR (dB)	−13.24	−13.578	−5.158
Range ISLR (dB)	−9.76	−9.77	−1.906

**Table 6:** Comparison of Computational Load.

Target	Proposed Algorithm	BPA	CSA
Computational load (GFLOP)	6790.6512	6,865,799.04	2415.32

**Figure C1:**
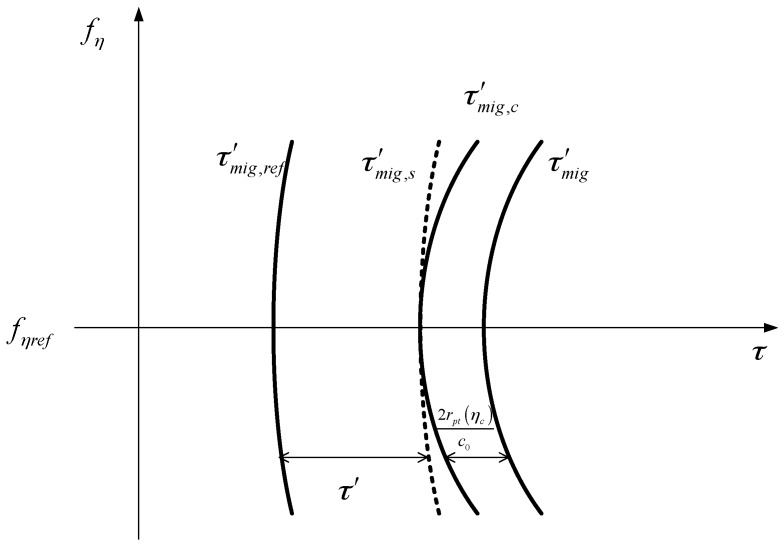
Illustration of important curves and variables in RD domain. $\tau_{mig,ref}^{\prime }$ and $\tau_{mig}^{\prime }$ represent migration curves corresponding to swath center and any target in the swath, respectively. $\tau_{mig,c}^{\prime }$ and $\tau_{mig,s}^{\prime }$ represent migration curves corresponding to any target whose $\eta_{c}=0$ before and after RCMC, respectively. $\tau^{\prime }$ is the difference between $\tau_{mig,s}^{\prime }$ and $\tau_{mig,ref}^{\prime }$ . $2r_{pt}\left(\eta_{c}\right)/c_{0}$ is the range offset induced by first azimuth scaling.

**Figure D1:**
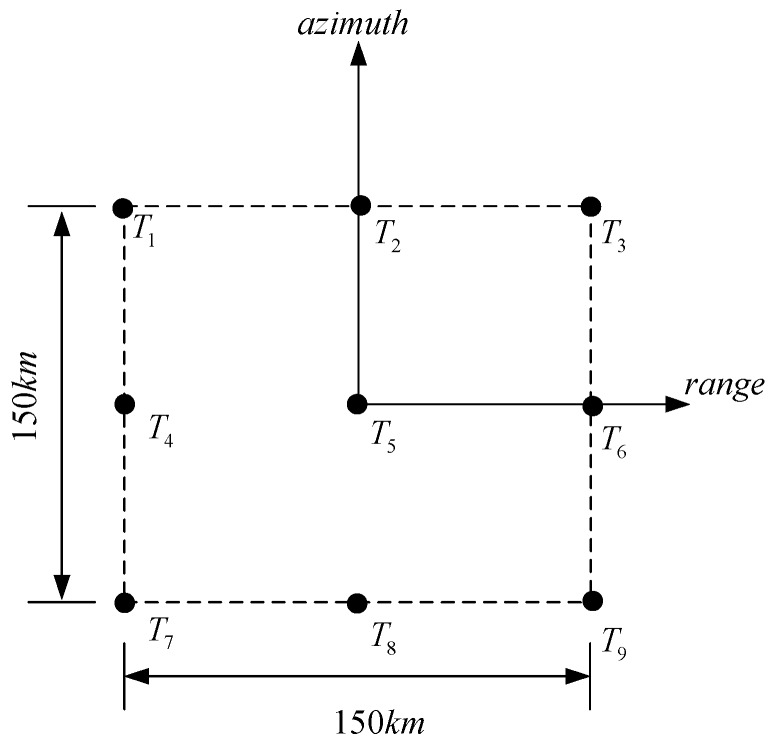
T_5_ is at swath center. T_1_ is 75 km away from T_5_ along the azimuth and range directions.

**Figure D2:**
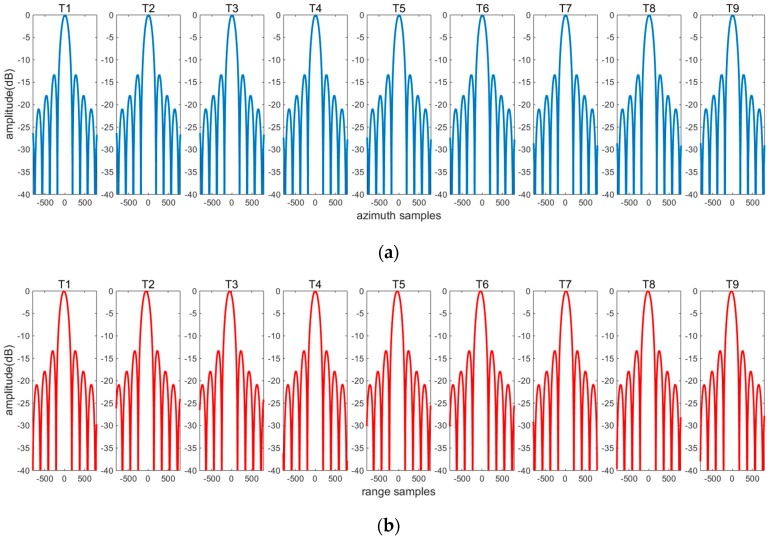
(**a**) Azimuth and (**b**) range profiles corresponding to every point target, represented by blue and red lines, respectively.

## Appendix A

Let $R_{t}\left(\eta \right)$ denote the one-way slant range between the satellite and target when transmitting a pulse, whose representation is:

(A1) $$R_{t}\left(\eta \right)=\sqrt{\langle {\vec{R}}_{g\_sat}\left(\eta \right)-{\vec{R}}_{g\_tar},{\vec{R}}_{g\_sat}\left(\eta \right)-{\vec{R}}_{g\_tar}\rangle }$$

where ${\vec{R}}_{g\_sat}\left(\eta \right)$ and ${\vec{R}}_{g\_tar}$ are position vectors of the satellite and the target in the Earth Centered Rotating (ECR) coordinate system [18], respectively. By applying series expansion, Equation (A1) can be expressed as:

(A2) $$R_{t}\left(\eta \right)=r_{0,\sin}+\sum_{n=1}^{\infty }\frac{r_{n,\sin}}{n!}{\left(\eta -\eta_{c}\right)}^{n}$$

$r_{n,\sin}$ denotes the nth-order coefficient. Furthermore, $R_{t}\left(\eta \right)$ can also be expressed as:

(A3) $$R_{t}\left(\eta \right)=\sqrt{{\left|{\vec{R}}_{g\_sat}\left(\eta \right)\right|}^{2}+{\left|{\vec{R}}_{g\_tar}\right|}^{2}-2\langle {\vec{R}}_{g\_sat}\left(\eta \right),{\vec{R}}_{g\_tar}\rangle }$$

Let $R_{r}\left(\eta +\Delta \eta \right)$ denote the one-way slant range when receiving the echo, where Δη denotes the round-trip delay time. For GEO SAR, the farthest slant range is about 41,680 km, which makes the round-trip delay time less than 0.28 s. As a result, the radius of the GEO SAR trajectory during the round-trip delay time is approximately invariable, which indicates $\left|{\vec{R}}_{g\_sat}\left(\eta +\Delta \eta \right)\right|\approx \left|{\vec{R}}_{g\_sat}\left(\eta \right)\right|$. By combining Equations (A1) and (A2), $R_{r}\left(\eta +\Delta \eta \right)$ equals:

(A4) $$\begin{array}{ll} R_{r}\left(\eta +\Delta \eta \right) & =\sqrt{{\left|{\vec{R}}_{g\_sat}\left(\eta +\Delta \eta \right)\right|}^{2}+{\left|{\vec{R}}_{g\_tar}\right|}^{2}-2\langle {\vec{R}}_{g\_sat}\left(\eta +\Delta \eta \right),{\vec{R}}_{g\_tar}\rangle }\\ & \approx \sqrt{R_{t}^{2}\left(\eta \right)-2\langle {\vec{R}}_{g\_sat}\left(\eta +\Delta \eta \right)-{\vec{R}}_{g\_sat}\left(\eta \right),{\vec{R}}_{g\_tar}\rangle } \end{array}$$

$R_{t}\left(\eta \right)$ and $R_{r}\left(\eta +\Delta \eta \right)$ also satisfies the following propagation equation:

(A5) $$R_{t}\left(\eta \right)+R_{r}\left(\eta +\Delta \eta \right)=c_{0}\Delta \eta$$

Combining Equations (A4) and (A5), the following equation can be achieved:

(A6) $$2\langle {\vec{R}}_{g\_sat}\left(\eta +\Delta \eta \right)-{\vec{R}}_{g\_sat}\left(\eta \right),{\vec{R}}_{g\_tar}\rangle +{\left(c_{0}\Delta \eta \right)}^{2}=2c_{0}\Delta \eta R_{t}\left(\eta \right)$$

The satellite position at η+Δη can be approximated as:

(A7) $${\vec{R}}_{g\_sat}\left(\eta +\Delta \eta \right)\approx {\vec{R}}_{g\_sat}\left(\eta \right)+{\vec{V}}_{g\_sat}\left(\eta \right)\Delta \eta +\frac{1}{2}{\vec{A}}_{g\_sat}\left(\eta \right){\left(\Delta \eta \right)}^{2}$$

where ${\vec{V}}_{g\_sat}\left(\eta \right)$ and ${\vec{A}}_{g\_sat}\left(\eta \right)$ are the velocity and acceleration vectors of the satellite, respectively. By replacing the component of ${\vec{R}}_{g\_sat}\left(\eta +\Delta \eta \right)-{\vec{R}}_{g\_sat}\left(\eta \right)$ in Equation (A6) with Equation (A7), Δη can be solved:

(A8) $$\Delta \eta =\frac{2c_{0}R_{t}\left(\eta \right)-2\langle {\vec{V}}_{sg}\left(\eta \right),{\vec{R}}_{g\_tar}\rangle }{c_{0}^{2}+\langle {\vec{A}}_{sg}\left(\eta \right),{\vec{R}}_{g\_tar}\rangle }$$

Since $c_{0}^{2}\gg \langle {\vec{A}}_{sg}\left(\eta \right),{\vec{R}}_{g\_tar}\rangle$, Δη can be approximated as:

(A9) $$\Delta \eta \approx \frac{2R_{t}\left(\eta \right)}{c_{0}}-\frac{2\langle {\vec{V}}_{sg}\left(\eta \right),{\vec{R}}_{g\_tar}\rangle }{c_{0}^{2}}-\frac{2\langle {\vec{A}}_{sg}\left(\eta \right),{\vec{R}}_{g\_tar}\rangle }{c_{0}^{3}}R_{t}\left(\eta \right)$$

At the beam crossing time $\eta_{c}$, the satellite position vector ${\vec{R}}_{g\_sat}\left(\eta \right)$ can be represented as:

(A10) $${\vec{R}}_{g\_sat}\left(\eta \right)={{\vec{R}}_{g\_sat}|}_{\eta =\eta_{c}}+\sum_{n=1}^{\infty }\frac{1}{n!}\cdot {{\vec{R}}_{g\_sat}^{\left(n\right)}|}_{\eta =\eta_{c}}\cdot {\left(\eta -\eta_{c}\right)}^{n}$$

where ${{\vec{R}}_{g\_sat}^{\left(n\right)}|}_{\eta =\eta_{c}}$ denotes the *n*th-order derivative of ${\vec{R}}_{g\_sat}\left(\eta \right)$ at $\eta_{c}$. Especially, ${{\vec{R}}_{g\_sat}^{\left(0\right)}|}_{\eta =\eta_{c}}$, ${{\vec{R}}_{g\_sat}^{\left(1\right)}|}_{\eta =\eta_{c}}$ and ${{\vec{R}}_{g\_sat}^{\left(2\right)}|}_{\eta =\eta_{c}}$ represent position vector, velocity vector and acceleration vector, respectively.

Because ${\vec{V}}_{g\_sat}\left(\eta \right)$ and ${\vec{A}}_{g\_sat}\left(\eta \right)$ are the first-order and second-order derivatives of ${\vec{R}}_{g\_sat}\left(\eta \right)$, so the following equations are achieved based on Equation (A10):

(A11) $$\{\begin{array}{l} {\vec{V}}_{sg}\left(\eta \right)=\sum_{n=1}^{\infty }\frac{1}{\left(n-1\right)!}\cdot {{\vec{R}}_{g\_sat}^{\left(n\right)}|}_{\eta =\eta_{c}}\cdot {\left(\eta -\eta_{c}\right)}^{n-1}\\ {\vec{A}}_{sg}\left(\eta \right)=\sum_{n=2}^{\infty }\frac{1}{\left(n-2\right)!}\cdot {{\vec{R}}_{g\_sat}^{\left(n\right)}|}_{\eta =\eta_{c}}\cdot {\left(\eta -\eta_{c}\right)}^{n-2} \end{array}$$

By substituting Equations (A2) and (A11) into Equation (A9), the equivalent slant range R(η) equals:

(A12) $$\begin{array}{ll} R\left(\eta \right) & =c_{0}\Delta \eta /2\\ & =r_{0,\sin}+\sum_{n=1}^{\infty }\frac{r_{n,\sin}}{n!}{\left(\eta -\eta_{c}\right)}^{n}-\frac{1}{c_{0}}\sum_{n=1}^{\infty }\frac{k_{n}}{\left(n-1\right)!}{\left(\eta -\eta_{c}\right)}^{n-1}\\ & -\left[\frac{1}{c_{0}^{2}}\sum_{n=2}^{\infty }\frac{k_{n}}{\left(n-2\right)!}{\left(\eta -\eta_{c}\right)}^{n-2}\right]\left[r_{0,\sin}+\sum_{n=1}^{\infty }\frac{r_{n,\sin}}{n!}{\left(\eta -\eta_{c}\right)}^{n}\right] \end{array}$$

where $k_{n}$ denotes $\langle {{\vec{R}}_{g\_sat}^{\left(n\right)}|}_{\eta =\eta_{c}},{\vec{R}}_{g\_tar}\rangle$.

Equation (A12) can be simplified as:

(A13) $$R\left(\eta \right)\approx r_{0}+\sum_{n=1}^{\infty }\frac{r_{n}}{n!}{\left(\eta -\eta_{c}\right)}^{n}$$

where:

(A14) $$r_{n}=r_{n,\sin}-\frac{k_{n+1}}{c_{0}}-\frac{1}{c_{0}^{2}}\cdot \sum_{m=0}^{n}C_{n}^{m}k_{m+2}r_{n-m,\sin},\;n\geq 0$$

and $C_{n}^{m}=n!/\left[(n-m)!m!\right]$.

## Appendix B

Applying azimuth FT on Equation (7), the integration phase is:

(B1) $$\theta_{\mathrm{int}}=-\frac{4\pi \left(f_{0}+f_{\tau }\right)}{c_{0}}R(\eta )-2\pi f_{\eta }\eta$$

According to the principle of stationary phase and Equation (2) with $N_{r}=5$, the following equation can be acquired from Equation (B1) by making the derivative of $\theta_{\mathrm{int}}$ be zero:

(B2) $$r_{2}\eta +\frac{1}{2}r_{3}\eta^{2}+\frac{1}{6}r_{4}\eta^{3}+\frac{1}{24}r_{5}\eta^{4}=-\frac{c_{0}f_{\eta }}{2\left(f_{0}+f_{\tau }\right)}$$

Based on the series reversion method, η can be expressed as:

(B3) $$\eta =A_{1}y+A_{2}y^{2}+\cdots +A_{9}y^{9}$$

where *y* denotes $-c_{0}f_{\eta }/\left[2\left(f_{0}+f_{\tau }\right)\right]$. $A_{1}$ through $A_{9}$ denote stationary phase point coefficients to be solved below.

Substituting Equation (B3) into Equation (B2), the following equation holds:

(B4) $$\begin{array}{l} r_{2}\left[A_{1}y+A_{2}y^{2}+\cdots +A_{9}y^{9}\right]+\frac{1}{2}r_{3}{\left[A_{1}y+A_{2}y^{2}+\cdots +A_{9}y^{9}\right]}^{2}+\cdots \\ +\frac{1}{4!}r_{5}{\left[A_{1}y+A_{2}y^{2}+\cdots +A_{9}y^{9}\right]}^{4}=y \end{array}$$

From Equation (B4), a set of coefficient equations is acquired as follows:

(B5) $$\{\begin{array}{l} r_{2}A_{1}=1\\ r_{2}A_{2}+\frac{1}{2}r_{3}A_{1}^{2}=0\\ r_{2}A_{3}+r_{3}A_{1}A_{2}+\frac{1}{6}r_{4}A_{1}^{3}=0\\ \vdots \end{array}$$

By solving Equation (B5), $A_{i}$ satisfies:

(B6) $$\left\{ \begin{array}{l} A_{1}=\frac{1}{r_{2}}\\ A_{2}=-\frac{r_{3}}{2r_{2}^{3}}\\ A_{3}=\frac{1}{6r_{2}^{5}}\left[3r_{3}^{2}-r_{2}r_{4}\right]\\ A_{4}=\frac{1}{24r_{2}^{7}}\left[10r_{2}r_{3}r_{4}-15r_{3}^{3}-r_{2}^{2}r_{5}\right]\\ A_{5}=\frac{1}{120r_{2}^{9}}\left[-r_{2}^{3}r_{6}+15r_{5}r_{2}^{2}r_{3}+10r_{2}^{2}r_{4}^{2}-105r_{2}r_{3}^{2}r_{4}+105r_{3}^{4}\right]\\ A_{6}=\cdots \end{array}\right.$$

Substituting Equation (B3) into Equation (B1), the spectrum phase in the two dimensional frequency domain is acquired:

(B7) $$\theta_{2df}(f_{\eta },f_{\tau })=-\frac{4\pi (f_{0}+f_{\tau })}{c_{0}}\left\{r_{0}-\sum_{n=1}^{9}\frac{A_{n}}{n+1}{\left(-\frac{c_{0}f_{\eta }}{2\left(f_{0}+f_{\tau }\right)}-r_{1}\right)}^{n+1}\right\}-\frac{\pi f_{\tau }^{2}}{K_{r}}-2\pi f_{\eta }\eta_{c}$$

By applying series expansion, Equation (B7) can be expressed as a polynomial of $f_{\tau }$:

(B8) $$\theta_{2df}(f_{\eta },f_{\tau })=2\pi \varphi_{0}+\sum_{k=1}^{9}\phi_{k}f_{\tau }^{k}-2\pi f_{\eta }\eta_{c}$$

where:

(B9) $$\left\{ \begin{array}{l} \varphi_{0}=-\frac{2}{\lambda }\left(r_{0}-P_{0}\right)+\sum_{m=1}^{10}\frac{2P_{m}}{\lambda }{\left(\frac{\lambda }{2}f_{\eta }\right)}^{m}\\ \phi_{1}=-\frac{2}{c_{0}}\left(r_{0}-P_{0}\right)-\sum_{m=2}^{10}\frac{2P_{m}}{c_{0}}{\left(\frac{\lambda }{2}f_{\eta }\right)}^{m}\left(m-1\right)\\ \phi_{2}=\sum_{m=2}^{10}\frac{2C_{m}^{2}P_{m}}{c_{0}f_{0}}{\left(\frac{\lambda }{2}f_{\eta }\right)}^{m}-\frac{1}{2K_{r}}\\ \phi_{k}={\left(-1\right)}^{k}\sum_{m=2}^{10}\frac{2C_{m+k-2}^{k}P_{m}}{c_{0}f_{0}^{k-1}}{\left(\frac{\lambda }{2}f_{\eta }\right)}^{m},k\geq 3 \end{array}\right.$$

and:

(B10) $$\left\{ \begin{array}{l} P_{0}=\sum_{n=1}^{9}\frac{A_{n}{\left(-r_{1}\right)}^{n+1}}{n+1}\\ P_{1}=\sum_{n=1}^{9}A_{n}{\left(-1\right)}^{n+1}r_{1}^{n}\\ P_{m}=\sum_{n=m-1}^{9}\frac{A_{n}{\left(-1\right)}^{n+1}C_{n+1}^{m}r_{1}^{n+1-m}}{n+1},2\leq m\leq 10 \end{array}\right.$$

Because $P_{m}$ depends on $r_{n}$ and the highest orders along range and azimuth are both 2 shown in Equation (6), $P_{m}$ can be modeled as:

(B11) $$P_{m}=P_{m,ref}+Q_{m,1,r}\Delta R+Q_{m,2,r}{\left(\Delta R\right)}^{2}+{Q_{m,1,a}|}_{\Delta R}\eta_{c}+{Q_{m,2,a}|}_{\Delta R}{\left(\eta_{c}\right)}^{2},0\leq m\leq 10$$

According to Equations (B6) and (B10), every coefficient in Equation (B11) can be obtained. Then coefficients in Equation (13) can be acquired:

(B12) $$\left\{ \begin{array}{l} \varphi_{0,ref}=-\frac{2}{\lambda }\left(r_{0,ref}-P_{0,ref}\right)+\sum_{m=1}^{10}\frac{2P_{m,ref}}{\lambda }{\left(\frac{\lambda }{2}f_{\eta }\right)}^{m}\\ \begin{array}{l} M_{1,r}=-\frac{2}{\lambda }\left(1-Q_{0,1,r}\right)+\sum_{m=1}^{10}\frac{2Q_{m,1,r}}{\lambda }{\left(\frac{\lambda }{2}f_{\eta }\right)}^{m}\\ M_{2,r}=-\frac{2}{\lambda }\left(-Q_{0,2,r}\right)+\sum_{m=1}^{10}\frac{2Q_{m,n,r}}{\lambda }{\left(\frac{\lambda }{2}f_{\eta }\right)}^{m}\\ \begin{array}{l} M_{1,a}=-\frac{2}{\lambda }\left(1-{Q_{0,1,a}|}_{\Delta R}\right)+\sum_{m=1}^{10}\frac{2{Q_{m,1,a}|}_{\Delta R}}{\lambda }{\left(\frac{\lambda }{2}f_{\eta }\right)}^{m}\\ M_{2,a}=-\frac{2}{\lambda }\left(-{Q_{0,2,a}|}_{\Delta R}\right)+\sum_{m=1}^{10}\frac{2{Q_{m,2,a}|}_{\Delta R}}{\lambda }{\left(\frac{\lambda }{2}f_{\eta }\right)}^{m} \end{array} \end{array} \end{array}\right.$$

(B13) $$\left\{ \begin{array}{l} \phi_{1,ref}=-\frac{2}{c_{0}}\left(r_{0,ref}-P_{0,ref}\right)-\sum_{m=2}^{10}\frac{2P_{m,ref}}{c_{0}}{\left(\frac{\lambda }{2}f_{\eta }\right)}^{m}\left(m-1\right)\\ \begin{array}{l} L_{1,r}=-\frac{2}{c_{0}}\left(1-Q_{0,1,r}\right)-\sum_{m=2}^{10}\frac{2Q_{m,1,r}}{c_{0}}{\left(\frac{\lambda }{2}f_{\eta }\right)}^{m}\left(m-1\right)\\ L_{2,r}=-\frac{2}{c_{0}}\left(-Q_{0,2,r}\right)-\sum_{m=2}^{10}\frac{2Q_{m,2,r}}{c_{0}}{\left(\frac{\lambda }{2}f_{\eta }\right)}^{m}\left(m-1\right)\\ L_{1,a}=-\frac{2}{c_{0}}\left(1-{Q_{0,1,a}|}_{\Delta R}\right)-\sum_{m=2}^{10}\frac{2{Q_{m,1,a}|}_{\Delta R}}{c_{0}}{\left(\frac{\lambda }{2}f_{\eta }\right)}^{m}\left(m-1\right) \end{array} \end{array}\right.$$

(B14) $$\{\begin{array}{l} \phi_{2,ref}=\sum_{m=2}^{10}\frac{2C_{m}^{2}P_{m,ref}}{c_{0}f_{0}}{\left(\frac{\lambda }{2}f_{\eta }\right)}^{m}-\frac{1}{2K_{r}}\\ \begin{array}{l} J_{1,r}=\sum_{m=2}^{10}\frac{2C_{m}^{2}Q_{m,1,r}}{c_{0}f_{0}}{\left(\frac{\lambda }{2}f_{\eta }\right)}^{m}\\ J_{1,a}=\sum_{m=2}^{10}\frac{2C_{m}^{2}{Q_{m,1,a}|}_{\Delta R}}{c_{0}f_{0}}{\left(\frac{\lambda }{2}f_{\eta }\right)}^{m} \end{array} \end{array}$$

(B15) $$\{\begin{array}{l} \phi_{3,ref}={\left(-1\right)}^{3}\sum_{m=2}^{10}\frac{2C_{m+1}^{3}P_{m,ref}}{c_{0}f_{0}^{2}}{\left(\frac{\lambda }{2}f_{\eta }\right)}^{m}\\ \begin{array}{l} K_{1,r}={\left(-1\right)}^{3}\sum_{m=2}^{10}\frac{2C_{m+1}^{3}Q_{m,1,r}}{c_{0}f_{0}^{2}}{\left(\frac{\lambda }{2}f_{\eta }\right)}^{m}\\ K_{1,a}={\left(-1\right)}^{3}\sum_{m=2}^{10}\frac{2C_{m+1}^{3}{Q_{m,1,a}|}_{\Delta R}}{c_{0}f_{0}^{2}}{\left(\frac{\lambda }{2}f_{\eta }\right)}^{m} \end{array} \end{array}$$

and:

(B16) $$\phi_{k,ref}={\left(-1\right)}^{k}\sum_{m=2}^{10}\frac{2C_{m+k-2}^{k}P_{m,ref}}{c_{0}f_{0}^{k-1}}{\left(\frac{\lambda }{2}f_{\eta }\right)}^{m},k\geq 4$$

## Appendix C

After multiplying Equation (34) with Equation (37), the two-dimensional spectrum is:

(C1) $$\begin{array}{ll} S_{2df}^{\prime \prime }\left(f_{\eta },f_{\tau }\right) & =\sigma \cdot W_{a}\left[f_{\eta }\right]\cdot rect\left[\frac{f_{\tau }}{B_{r}}\right]\exp\left\{j2\pi \sum_{k=1}^{3}\phi_{k}^{\prime }f_{\tau }^{k}\right\}\exp\left\{j2\pi \varphi_{0}^{\prime }\right\}\\ & \cdot \exp\left\{j\frac{2\pi }{3}Yf_{\tau }^{3}\right\}\exp\left\{-j2\pi f_{\eta }\eta_{c}\right\} \end{array}$$

Implementing range IFT on it, the signal in the RD domain is obtained by the series reversion method:

(C2) $$\begin{array}{l} S_{rd}\left(f_{\eta },\tau \right)=rect\left[\frac{\tau -\tau_{mig}^{\prime }}{T_{p}}\right]\cdot \exp\left\{j2\pi \varphi_{0}^{\prime }\right\}\cdot \exp\left\{-j2\pi f_{\eta }\eta_{c}\right\}\\ \cdot \exp\left\{-j\pi K_{m}\cdot {\left[\tau -\tau_{mig}^{\prime }\right]}^{2}\right\}\cdot \exp\left\{-j\frac{2\pi }{3}J_{m}\cdot {\left[\tau -\tau_{mig}^{\prime }\right]}^{3}\right\} \end{array}$$

where ${\tau^{\prime }}_{mig}=-\phi_{1}^{\prime }$, $K_{m}=1/\left(2\phi_{2}^{\prime }\right)$ and $J_{m}=\left(3\phi_{3}^{\prime }+Y\right)K_{m}^{3}$.

Some important curves and variables in the RD domain are illustrated in Figure C1, where:

(C3) $$\tau^{\prime }={\tau_{mig}^{\prime }|}_{f_{\eta }=f_{\eta ref}}-\frac{2r_{pt}\left(\eta_{c}\right)}{c_{0}}-{\tau_{mig,ref}^{\prime }|}_{f_{\eta }=f_{\eta ref}}$$

Considering the spatial variance model in Equation (35), we can represent $\tau^{\prime }$ as:

(C4) $$\tau^{\prime }=-L_{1,r,ref}^{\prime }\Delta R-L_{2,r,ref}^{\prime }{\left(\Delta R\right)}^{2}$$

Combining Equations (35) and (C4), the differential RCM of the range cell center defined as $\tau_{mig,c}^{\prime }-\tau_{mig,s}^{\prime }$ is obtained:

(C5) $$\frac{2}{c_{0}}RCM_{diff}=\alpha \cdot \tau^{\prime }+\beta \cdot {\left(\tau^{\prime }\right)}^{2}$$

where:

(C6) $$\left\{ \begin{array}{l} \alpha =\frac{L_{1,r}^{\prime }}{L_{1,r,ref}^{\prime }}-1\\ \beta =\frac{L_{1,r}^{\prime }L_{2,r,ref}^{\prime }-L_{2,r}^{\prime }L_{1,r,ref}^{\prime }}{{\left(L_{1,r,ref}^{\prime }\right)}^{3}} \end{array}\right.$$

$L_{1,r,ref}^{\prime }$ and $L_{2,r,ref}^{\prime }$ equal ${L_{1,r}^{\prime }|}_{f_{\eta }=f_{\eta ref}}$ and ${L_{2,r}^{\prime }|}_{f_{\eta }=f_{\eta ref}}$. Then, based on Equation (C5), $\tau -\tau_{mig}^{\prime }$ can also be represented as a function about $\tau^{\prime }$:

(C7) $$\tau -{\tau^{\prime }}_{mig}=\tau_{1}-t_{con}-\alpha \cdot \tau^{\prime }-\beta \cdot {\left(\tau^{\prime }\right)}^{2}$$

where $\tau_{1}=\tau -\tau_{mig,s}^{\prime }$.

$K_{m}$ and $J_{m}$ can also be represented as functions of $\tau^{\prime }$:

(C8) $$\{\begin{array}{l} K_{m}=K_{mref}+K_{s}\tau^{\prime }\\ J_{m}=J_{mref}+J_{s}\tau^{\prime } \end{array}$$

where:

(C9) $$\left\{ \begin{array}{l} K_{mref}=\frac{1}{2\phi_{2,ref}^{\prime }}\\ K_{s}=\frac{2K_{mref}^{2}J_{1,r}^{\prime }}{L_{1,r}^{\prime }}\\ J_{mref}=\left(3\phi_{3,ref}^{\prime }+Y\right)K_{mref}^{3}\\ J_{s}=-\frac{3K_{1,r}^{\prime }K_{mref}^{3}}{L_{1,r,ref}^{\prime }}+\frac{6J_{1,r}^{\prime }K_{mref}J_{mref}}{L_{1,r,ref}^{\prime }} \end{array}\right.$$

Based on Equations (C7) and (C8), the signal in Equation (C2) can be represented as Equation (38).

## Appendix D

This appendix will show the swath size achieved by the proposed algorithm can reach 150 km (azimuth) × 150 km (range). Simulation parameters are listed in Table D1. The simulated swath is portrayed in Figure D1, and the swath center is at the equator.

**Table D1:** Parameters for Simulation and Verification.

Parameters	Value
Orbital inclination angle	60°
Eccentricity	0
Center time	0 s
Wavelength	0.24 m
Pulse width	2 μs
Bandwidth	150 MHz
Sampling rate	250 MHz
Pulse repetition frequency	400 Hz
Incidence angle	35°
Squint angle	90°
Synthetic aperture time	630 s

Range and azimuth profiles corresponding to every target are illustrated in Figure D2, and evaluation results are listed in Table D2. As shown in Table D2, the difference of range PSLRs, azimuth PSLRs, range ISLRs, and azimuth ISLRs over the whole swath is ≤0.02 dB, ≤0.06 dB, ≤0.03 dB, and ≤0.09 dB respectively. Some broadening coefficients are less than 1, because the modulation rates corresponding to these points are changed in imaging, and the resolutions become better. The simulation results show that this algorithm can achieve good imaging quality over the swath of 150 km × 150 km.

**Table D2:** Evaluation Results.

Target	T_1_	T_2_	T_3_	T_4_	T_5_	T_6_	T_7_	T_8_	T_9_
Azimuth resolution (m)	1.95	1.93	1.95	1.96	1.95	1.96	1.98	1.98	1.97
Azimuth broadening	0.99	1	0.99	0.98	1	0.99	0.98	1	1
Azimuth PSLR (dB)	13.28	−13.27	−13.30	−13.28	−13.28	−13.29	−13.28	−13.28	−13.24
Azimuth ISLR (dB)	−10.18	−10.21	−10.26	−10.17	−10.17	−10.24	−10.18	−10.17	−10.19
Range resolution (m)	2.09	2.09	2.08	2.07	2.07	2.06	2.08	2.06	2.06
Range broadening	0.98	1	0.99	0.98	1	0.98	1	1	0.98
Range PSLR (dB)	−13.25	−13.25	−13.25	−13.25	−13.25	−13.25	−13.26	−13.25	−13.24
Range ISLR (dB)	−10.16	−10.15	−10.15	−10.14	−10.15	−10.14	−10.15	−10.17	−10.14

## References

[1] Tomiyasu K. Synthetic aperture radar in geosynchronous orbit. Proceedings of the Antennas and Propagation Society International Symposium.

[2] Bruno D., Hobbs S.E., Ottavianelli G. (2006). Geosynchronous synthetic aperture radar: Concept design, properties and possible applications. Acta Astronaut..

[3] Tomiyasu K., Pacelli J.L. (1983). Synthetic aperture radar imaging from an inclined geosynchronous orbit. IEEE Trans. Geosci. Remote Sens..

[4] Hobbs S.E., Bruno D. Radar imaging from GEO: Challenges and applications. Proceedings of the Remote Sensing and Photogrammetry Society Annual Conference.

[5] Global Eartquake Satellite System (GESS). http://solidearth.jpl.nasa.gov/gess.html.

[6] Guarnieri A.M., Tebaldini S., Rocca F., Broquetas A. Gemini: Geosynchronous SAR for earth monitoring by interferometry and imaging. Proceedings of the 2012 IEEE International Geoscience and Remote Sensing Symposium.

[7] Schmidt A.R. (1986). Secondary Range Compression for Improved Range Doppler Processing of SAR Data with Highsquint. Master’s Thesis.

[8] Davison G.W., Cumming I.G., Ito M.R. (1996). A Chirp Scaling Approach for processing squint mode SAR data. IEEE Trans. Aerosp. Electron. Syst..

[9] Cafforio C., Prati C., Rocca F. Full resolution focusing of SEASAT SAR images in the frequency-wave number domain. Proceedings of the 8th EARSel Workshop.

[10] Zeng T., Li Y., Ding Z., Long T., Yao D., Sun Y. (2015). Subaperture approach based on azimuth-dependent range cell migration correction and azimuth focusing parameter equalization for maneuvering high-squint-mode SAR. IEEE Trans. Geosci. Remote Sens..

[11] Bao M., Xu G., Li Y.C., Xing M.D., Zheng B., Wang W. (2011). Research on curve trajectory model and imaging algorithm for GEO SAR. J. Astronaut..

[12] Bao M., Xing M.D., Li Y.C. (2012). Chirp scaling algorithm for GEOSAR based on fourth-order range equation. Electron. Lett..

[13] Hu B., Jiang Y.C., Zhang S.S., Yeo T.S. (2015). Focusing of geosynchronous SAR with nonlinear chirp scaling algorithm. Electron. Lett..

[14] Sun G.C., Xing M.D., Wang Y., Yang J., Bao Z. (2014). A 2-D space-variant chirp scaling algorithm based on the RCM equalization and subband systhesis toprocess geosynchronous SAR data. IEEE Trans. Geosci. Remote Sens..

[15] Hu B., Jiang Y., Zhang S., Zhang Y., Yeo T.S. (2015). Generalized Omega-K Algorithm for Geosynchronous SAR Image Formation. IEEE Geosci. Remote Sens. Lett..

[16] Ding Z., Shu B., Yin W., Zeng T., Long T. (2016). A Modified Frequency Domain Algorithm Based on Optimal Azimuth Quadratic Factor Compensation for Geosynchronous SAR Imaging. IEEE J. Sel. Top. Appl. Earth Observ. Remote Sens..

[17] Li D., Wu M., Sun Z., He F., Dong Z. (2015). Modeling and processing of two-dimensional spatial-variant geosynchronous SAR data. IEEE J. Sel. Top. Appl. Earth Observ. Remote Sens..

[18] Cumming I.G., Wong F.H. (2005). Digital Processing of Synthetic Aperture Radar Data: Algorithms and Implementation.

[19] Pei L., Gao L.N., Dong X.C., Long T. (2010). High accurate compensation method of Doppler center frequency in GEO SAR using phase scan. Trans. Beijing Inst. Technol..

[20] Tom M.A. (2004). Mathematical Analysis.

[21] Neo Y.L., Wong F.H., Cumming I.G. (2007). A two-dimentional spectrum for bistatic SAR processing using series reversion. IEEE Geosci. Remote Sens. Lett..

[22] Curlander J., McDonough R. (1991). Synthetic Aperture Radar: Systems and Signal Processing.

[23] Li Z., Li C.S., Yu Z., Zhou J., Chen J. Back projection algorithm for high resolution GEO-SAR imaging formation. Proceedings of the 2011 IEEE International on Geoscience and Remote Sensing Symposium (IGARSS).

[24] Wong F.H., Yeo T.S. (2001). New applications of nonlinear chirp scaling in SAR data processing. IEEE Trans. Geosci. Remote Sens..

